# State-of-the-Art Review on the Behavior of Bio-Asphalt Binders and Mixtures

**DOI:** 10.3390/molecules29163835

**Published:** 2024-08-13

**Authors:** Ghazi G. Al-Khateeb, Sara A. Alattieh, Waleed Zeiada, Cassie Castorena

**Affiliations:** 1 Department of Civil and Environmental Engineering, University of Sharjah, Sharjah P.O. Box 27272, United Arab Emirates; u20106073@sharjah.ac.ae (S.A.A.); wzeiada@sharjah.ac.ae (W.Z.); 2 Civil Engineering Department, Jordan University of Science and Technology, Irbid 3030, Jordan; 3 Department of Public Works Engineering, College of Engineering, Mansoura University, Mansoura 35516, Egypt; 4 Department of Civil, Construction, and Environmental Engineering, North Carolina State University, Raleigh, NC 27695, USA; cahintz@ncsu.edu

**Keywords:** asphalt pavements, renewable materials, bio-oil, bio-binders, asphalt performance, sustainability

## Abstract

Asphalt binder is the most common material used in road construction. However, the need for more durable and safer pavements requires a better understanding of asphalt’s aging mechanisms and how its characteristics can be improved. The current challenge for the road industry is to use renewable materials (i.e., biomaterials not subjected to depletion) as a partial replacement for petroleum-based asphalt, which leads to reducing the carbon footprint. The most promising is to utilize biomaterials following the principles of sustainability in the modification of the asphalt binder. However, to understand whether the application of renewable materials represents a reliable and viable solution or just a research idea, this review covers various techniques for extracting bio-oil and preparing bio-modified asphalt binders, technical aspects including physical properties of different bio-oils, the impact of bio-oil addition on asphalt binder performance, and the compatibility of bio-oils with conventional binders. Key findings indicate that bio-oil can enhance modified asphalt binders’ low-temperature performance and aging resistance. However, the effect on high-temperature performance varies based on the bio-oil source and preparation method. The paper concludes that while bio-oils show promise as renewable modifiers for asphalt binders, further research is needed to optimize their use and fully understand their long-term performance implications.

## 1. Introduction

In the last few decades, researchers in asphalt technology have begun exploring alternatives to asphalt binders derived from fossil fuel as innovative and environmentally friendly materials for pavements. These products can be used in hot mix asphalt (HMA) to potentially mitigate the escalating costs of asphalt binders and decrease the demand for petroleum-derived products [1,2,3]. Strategies employed to mitigate these factors include the use of natural asphalt extracted from natural sources, such as the natural bitumen reserves in Canada, Venezuela, and Russia or Trinidad Lake asphalt (TLA), and increasing the use of recycled materials, including Reclaimed Asphalt Pavement (RAP), Reclaimed Asphalt Shingles (RAS), or hard asphalt particles (HAP) [4,5,6,7,8,9,10,11]. Various additives have changed and enhanced asphalt binder characteristics, including traditional modifiers such as nano-materials, polymers, rubber powder, and Poly(styrene-butadiene-styrene) (SBS). Additionally, waste mitigation efforts have incorporated waste materials such as ash, plastic waste, and other recyclable substances [12,13,14,15,16]. 

Biomaterials are considered one of the materials that can partially replace or modify petroleum-based asphalt and are produced from different biomass sources or waste materials. Biomaterials, including bio-binders and bio-oils, were widely investigated in the literature [5,17]. Bio-oils have been proposed to be produced purposefully for use in pavements. In contrast, others have been proposed to be considered waste materials, thus reusing them in pavements will benefit the environment and promote sustainability. Bio-oil/bio-binder (BB) can be classified as either the product of transforming biomass (i.e., manure, wood, waste materials) through several conversion techniques into oil or the use of oil products such as waste cooking oil (WCO), waste engine oil (WEO), vegetable oil, etc. [18,19]. The bio-modified asphalt binder (BMB) is referred to as the product of the modification of asphalt binder with bio-oil [20]. In addition, bio-oils used for bitumen fluxing show significant potential in RAP technology, enhancing the blending of RAP with virgin materials and rejuvenating the binder in RAP [21,22].

The expense linked to the creation of bio-oil adjusted asphalt binders is contingent upon factors like the origin and type of bio-oil, the method of preparation, and the performance criteria. According to a recent investigation, the existing expense for manufacturing one metric ton of bio-binder in China ranges between CNY 1500 and 2000, notably lower than petroleum-based asphalt, which commands roughly CNY 3000 to 5000 per ton [2]. In a separate inquiry, it was observed that bio-asphalt, when enhanced with 10% bio-oil and styrene-butadiene-styrene (SBS), could viably replace 50 penetration grade petroleum asphalt binder in road functionality, at a more economical price [23]. Nevertheless, the expense associated with bio-oil-modified asphalt binders can also fluctuate depending on factors such as the quality and availability of biomass resources, as well as the costs linked with transportation, storage, and adherence to environmental regulations. Notably, estimations propose a potential reduction of approximately 25–30% in the production cost of bio-binders compared to petroleum-based asphalt [24,25].

Several research studies have used bio-oils and produced so-called bio-binders in HMAs. Bio-binders have generally been produced from waste bio-oils; hence, this activity serves multiple goals: (1) using bio-oils to produce sustainable and less costly binders, (2) reducing the negative impact that waste oils can cause if not properly disposed of and recycled to keep the environment clean, and (3) reducing reliance on the natural resources of oil. However, questions remain pertaining to whether bio-oils are suitable for use in asphalt production and construction: do they require changes to the typical production processes for asphalt mixtures, can the current specifications for asphalt binders be used to ‘screen’ and accept bio-asphalts, and what are the optimum contents to be used?

This overview paper sheds light on previous research studies related to this topic. The objective of this paper is to identify the main sources of biomass used to produce bio-oils, extraction techniques, and mixing conditions, in addition to a comprehensive assessment of BMB blends from different studies. A summary of conclusions and findings of past studies that proposed and evaluated bio-oil as a modifier for asphalt binders is presented in this paper. Important findings of the collective literature are discussed, including areas where the results of past studies are contradictory. Some recommendations are given at the end of the overview to guide future research in this field by suggesting key focus areas. This review covers the main sources of bio-oils, extraction, modification techniques of asphalt binder, and the performance characterization of BMB mixtures. The various types of bio-oils used in asphalt modification and mixing methods used to achieve compatibility and homogenous blends are presented. This paper highlights the effect of bio-oil on the rheological properties and performance of asphalt binders and asphalt mixtures. 

## 2. Main Sources of Bio-Binder

According to the research studies in the literature, several main sources of bio-oils have been used for asphalt binders and mixtures as alternatives to crude oil sources. The bio-oil sources discussed in the literature are generally considered waste biomaterials. Common alternative binders that have been used by other researchers include bio-oils produced from swine waste or waste wood, waste cooking oil, vegetable oils, and pyrolyzed materials [26]. A discussion of bio-oils derived from each of these sources is provided below:

Multiple research studies were conducted to evaluate the performance of BB produced from swine manure. Those studies evaluated the potential of alternating the crude oil source for asphalt binder with the binder produced from swine manure conversion at different oil contents, reaching a 100% replacement. Another widely used source in the literature is the bio-oil derived from waste wood. Three types of bio-oils derived from trees have been generated: untreated bio-oil, treated bio-oil, and polymer-modified bio-oil [27,28,29,30,31].

WCO is generated from frying activities at high temperatures in fast-food restaurants, food industries, hotels, and homes. If not properly managed and disposed of, the abundant production of WCO can significantly harm the environment. Recycling or reusing WCO in modified asphalt binder is, therefore, an effective and environmentally beneficial solution [32,33,34,35,36,37]. Additional types of bio-oils that are derived from vegetable sources such as soybean, rapeseed, castor, palm, cotton, date seed, etc., were used as viable sources of bio-oil.

Researchers considered different sources of BB, especially those that are bio-based sources or from waste materials such as WEO, resin, liquid (Dammar), and lignins as asphalt modifiers [38,39,40,41]. Further studies included the conversion of waste material such as the bio-fuel derived from refinery by-products or forest products into bio-oils [42]. 

## 3. Production Techniques of Bio-Binder/Bio-Oil

### 3.1. Bio-Binder/Oil Extraction

Biomass can be converted into various forms of energy through numerous technical processes, depending on the raw material characteristics and the desired energy type. A wide range of conversion techniques has been developed [20]. Many advanced technologies have been implemented to generate bio-oil from biomass materials. The most effective methods involve thermochemical processes, which heat organic materials in the absence of air to transform them into solids, bio-oil, and gases [43]. The following section explores the different techniques used in the literature to produce bio-binders or bio-oils.

#### 3.1.1. Thermochemical Conversion Technologies

Multiple techniques were used to produce bio-binders originating from different biomass sources to resemble the characteristics of petroleum asphalt. Different thermochemical conversion methods are used to convert biomass into bio-oil/binder. Hydrothermal liquefaction and fast pyrolysis techniques have been used extensively to produce bio-oil/bio-binder and have been proven to be highly effective due to the high yield rate of bio-oils [19]. The thermochemical conversion of biomass is an expedited and controlled process that simulates the natural production of fossil fuel. Fossil fuels (crude oil) are made from decomposing living organisms in the Earth’s crust. The fossil material is subjected to increased heat and pressure as it is buried underground. As the heat rises, the molecules begin to break apart [44]. The initial breakdown creates partially changed materials. The crude oil undergoes a certain procedure of refining, desalting, and distilling to produce petroleum-based asphalt binder [19].

Thermochemical technologies such as hydrothermal liquefaction and fast pyrolysis work by converting feedstock organic compounds into bio-oil products using heat. The hydrothermal liquefaction process is conducted under elevated pressure and temperature to keep water in either a liquid or supercritical state. In contrast, fast pyrolysis relies on considerably higher temperatures at atmospheric pressure. The biomass in the fast pyrolysis technique is rapidly heated with the absence of oxygen to fracture the material into vapors, aerosols, and bio-chars [19]. One aspect that can be mentioned is that thermochemical conversion of biomass often leads to multiple products, in most cases bio-char and bio-oil [45,46]. The optimum separation and extraction procedure for products obtained from hydrothermal biomass processing. The hydrothermal conversion of biomass involves a series of separation and extraction procedures to obtain various products. Initially, biomass and water are subjected to a hydrothermal process, producing gases and a mixture of solid and liquid products. The solid products undergo filtration and are then washed with acetone. After a second filtration, the solid products are divided into heavy bio-oil (the acetone-soluble fraction) and bio-char (the acetone-insoluble fraction). Concurrently, the liquid products are filtered and extracted with ether, resulting in the separation of light bio-oil (the ether-soluble fraction) and processed water. This methodical approach allows for the efficient extraction and separation of gases, bio-oils, bio-char, and processed water from the biomass conversion process [46]. A summary of both processes is presented in Table 1 [19].

Different studies showed that the preparation temperature and pressure should be over 300 °C and 10 MPa, respectively, for maximum yield of by-products in the conversion of biomass through pyrolysis [47]. In addition, the particle size has a direct impact on the liquid yield; smaller particles produce a higher liquid output [48]. In a review, researchers stated that different biomass sources can be utilized to produce the bio-oil/bio-binder as an alternative source to crude oil [45,46]. Biomass that could be used to produce bio-oil comes from various sources, including municipal waste, wood, animal excrement, and crop straw. However, optimal conditions depend on the feedstock. It was determined that the oil yield from the pyrolysis is at least 50–60% of the total mass of the feedstock [19,24]. The conversion of biomass into bio-oil through pyrolysis involves several steps. Biomass is first dried and ground into finer particles, which are then fed into a reactor with fluidizing gas for pyrolysis. The resulting mixture of gases, char, and bio-oil exits the reactor and enters a cyclone, where char is separated and collected. The remaining gas and vapor mixture is cooled in a condenser, separating bio-oil from the gas. The bio-oil is collected, and the gas is recycled back into the system to sustain the process. This procedure efficiently converts biomass into bio-oil while managing by-products like char and reusable gas [49].

Thermochemical conversion of swine manure waste and other algae types was investigated [47,50,51]. A fractionation process was conducted to extract water, solid residue, and some of the organic compounds from the bio-oil, producing a black sticky residue and was used as a replacement for asphalt [25,52,53]. Bio-oil has been extracted from the palm oil empty fruit bunch through fast pyrolysis, followed by a purification process to remove water from the oil in one study [54]. Another study was conducted to produce bio-oil from domestic waste [55]. Different studies generated bio-oil from waste woods [56,57]. However, even though these procedures are so far the only commercially practiced technology, it was found that the quality of the resulting bio-oil was relatively poor compared to heavy petroleum fuel due to the high oxygen/water contents, viscosity, and corrosiveness [58]. The high oxygen content accelerates the aging rate of the BMB [24,59]. The generated bio-oil needs to be upgraded/modified through different processes such as hydrotreating, hydrocracking, supercritical fluids extraction, solvent addition/esterification, emulsification, steam reforming, and chemical extraction to resemble the original characteristics of the petroleum fuel [19,60].

#### 3.1.2. Solvent Extraction

Solvent extraction is one of the traditional techniques of extracting oil from seeds [61] such as soybeans [62], corn, rapeseed [63], cotton [35], date seeds [64,65], castor, and Pongamia [66], in addition to other sources such as algae [51,67]. Extraction via the Soxhlet apparatus is the most common method, which serves to extract the fats/lipids from biomass sources. In this method, the feedstock is placed in contact with a suitable solvent for extracting the oil from the solid particles to the liquid phase. The Soxhlet apparatus consists of several key components: a condenser at the top, an extraction chamber containing a thimble for holding the solid sample, a siphon arm for transferring the solvent, and a boiling flask at the bottom that holds the extraction solvent. As the solvent vaporizes, it travels up into the condenser, condenses, and drips into the extraction chamber. The solvent then extracts the desired compounds from the sample in the thimble. Once the solvent reaches a certain level, it siphons back into the boiling flask, carrying the extracted compounds with it, allowing for continuous extraction [68]. The solvent extraction technique is one of the cheapest and most efficient processes to obtain oil from seeds. However, the nature of the seed plays a major role in the amount of produced oil. The extraction solvent is heated under the bottom flask, which vaporizes into the sample thimble, condenses in the condenser, and drips back. When the liquid reaches the siphon arm, the liquid contents are emptied into the bottom flask again, and the process continues. This method requires a small quantity of the solvent. However, Soxhlet extraction has disadvantages, such as exposure to hazardous organic solvents that produce potentially toxic emissions during extraction. Solvents used in the extraction system must be of high purity, which can be costly. This procedure is not considered environmentally friendly and may contribute to pollution. The ideal sample for Soxhlet extraction is also limited to a dry and finely divided solid, and many factors, such as temperature and solvent–sample ratio, need to be considered when applying this method [68]. Bio-derived oil using the Soxhlet apparatus are usually light in color and weight compared to bio-oils generated through hydrothermal activities [69].

#### 3.1.3. Other Techniques

Other researchers produced a controllable source for WCO through continuous heating of fresh soybean oil; however, the quality of the waste oil is uncontrollable so the experimental results are uncertain [70]. The soybean oil underwent a long heating time, up to 16 h at a temperature of 270 °C and a stirring rate of 1200 rpm, to produce a waste bio-oil with controllable viscosity, density, acid value, impurity content, and water contents. The viscosities of the heated oils were found to increase as the heating time increased. The consistency of the WCO was evaluated to further assess the performance of untreated and treated WCO bio-oil-modified asphalt binder [32]. Untreated WCO, collected from local restaurants, is often disposed of without any proper treatment. Chemical treatment, i.e., the transesterification process, is conducted to chemically react untreated WCO with methanol (alcohol) in the presence of sodium hydroxide (NaOH) as base catalysts to produce treated WCO.

### 3.2. Production of Bio-Modified Binder (BMB) and Asphalt Mixtures

#### 3.2.1. Classification and Application of Bio-Oils in Asphalt Pavement

BMB is classified as per the content of the bio-oil. A direct alternative to the asphalt binder, i.e., 100% bio-oil content, where the asphalt binder is fully replaced by oil, is one classification. Partial replacement of the asphalt binder is where the bio-oil content is between 25–75% of the total binder content, in which case the bio-oil is classified as an extender. The bio-oil is classified as a rejuvenating agent/modifier for aged or nonaged binders (e.g., RAP and RAS) where the bio-oil content is less than 10% of the total binder and different additives could be added to the blend for further improvements [24,71,72]. It was found that the use of direct alternatives cannot be achieved due to the limitations of the performance of current bio-oils, while the potential for using bio-oils as extenders is more viable. Alternative binders can be made more reliable with the addition of some enhancing additives [24]. The bio-oil contents in the studied literature varied as low as 0% and as high as 100%. However, most researchers have used bio-oil as an extender [72]. Some studies suggested the use of bio-oils as rejuvenators for RAP and/or RAS [4,5], which are extracted from asphalt pavements (asphalt mixtures or roads). Table 2 shows an example of recycled blends used in a study [73].

Reusing RAP in recycled pavements offers an alternative solution for reducing waste generated from road construction materials. This practice encompasses both environmental and cost benefits, contributing to sustainable construction practices by lowering material costs, reducing landfill use, and decreasing the demand for non-renewable resources [74,75]. Increasing the RAP content in HMA significantly reduces the total cost per ton of asphalt, particularly in the expenses associated with binder, aggregate, and burner fuel, while also addressing additional factors such as pollution control, rejuvenator, testing, and RAP processing [75]. On the other hand, the expected service life of the pavement will decrease if the pavement or asphalt mixture is poorly designed, which may lead to higher maintenance costs, energy consumption, and emissions. Utilizing high amounts of RAP material in road pavement raises the risk of premature pavement distress, such as fatigue cracking, due to the oxidized and embrittled asphalt within the recycled material. Rejuvenators are products intended to restore the properties of recycled asphalt and must satisfy both short-term and long-term criteria. The most common way to use rejuvenating agents is by mixing the rejuvenator with the virgin binder, then combining the recycled material and virgin aggregate in the mix at high temperatures. 

#### 3.2.2. Mixing Techniques for Bio-Modified Asphalt

In addition to determining the oil content, mixing asphalt and bio-oils has received considerable attention. A review paper [24] discussed the production of bio-asphalt. The researchers stated two steps to producing bio-binder: (1) heating up the asphalt matrix and (2) mixing the bio-oil with the asphalt binder at proper conditions, including mixing equipment, time, temperature, and shearing rate. The mixing temperature generally varies from 130 °C to 150 °C, which is selected relative to the mixing temperature of the asphalt mixtures. On the other hand, there was a wide variation in the mixing times reported in the literature. It is worth mentioning that long mixing duration at high temperatures and speeds used in high-shear mixers has been reported to induce aging in some studies [21,64,76] based on an observed reduction in the penetration of the modified asphalt binder. The optimum combination of these parameters (i.e., mixing time, temperature, and oil content) was assessed in another study [77]. The optimum values correspond to the minimum difference in the softening point. Four oil contents, temperatures, and mixing time design matrix were conducted at a constant shearing speed of 1500 rpm. It was found that the minimum softening point difference corresponded to a mixing duration of 2 h, a temperature of 220 °C, and 6% oil residue content. The mixing conditions were selected to minimize the softening point difference between the different asphalt blends. Mixing temperature and oil content are statistically more significant than mixing time, as indicated by the higher F-values for mixing temperature and oil content than those for mixing time. Table 3 summarizes the mixing procedures used in a broad set of studies in the literature to emphasize the variation across studies. 

In summary, the literature has demonstrated that the short- and long-term effectiveness of bio-oils in recycled binder blends and asphalt mixtures is influenced by several design factors, including the type, source, and quantity of recycled materials, as well as the source and type of virgin asphalt binder. Additionally, the efficacy of the recycling agent is affected by production factors such as mixing time, production temperature, and the method of incorporating bio-oil into the asphalt binder or mixture. Therefore, it is crucial to consider the appropriate selection and combination of materials when producing recycled asphalt mixtures with high quantities of recycled materials. Practice-ready guidelines are needed for material selection and optimization, design, evaluation, and production of these mixtures [78]. The performance of the asphalt binder blended with different recycling agents was assessed through multiple criteria in the literature. One of these methods is based on the restoration of the performance grade (PG) of the recycled blends (virgin binder, recycled binder, and recycling agent) to determine the optimum recycling agent dosage [79]. Other research used penetration and ductility as the main indicators to study the bio-asphalt binders [21]. However, most of the literature was based on the performance of asphalt binders using multiple tests, as seen in the next part of this paper.

**Table 3:** Preparation parameters of bio-binder in the literature.

Author	Mixing Type	Oil Contents (%)	Mixing Conditions		
			Temperature (°C)	Duration (Min)	Speed (RPM)
[64]	High Shear Mixer	0, 1.5, 2.5, 3.5, 4.5, 5.5	150	60	4000
[80]		1, 2, 4, 6, 8	140–145	5	1600
[76]	Propeller	1, 2, 3, 4, 5	160	30	200
[81]		5, 10, 15, 20	135	15	3000
[82]	Mechanical	1, 2	150	6	2000
[83]	Propeller	3, 4, 5, 6, 7	130	15	1200
[35]		0, 5, 10	145	15	200
[1]	High Shear Mixer	3, 5, 10, 15, 20	135	30	3000
[84]	High Shear Mixer	10, 15	135	30	3000
[85]	Shear Mill	0.75	140	60	3000
[86]	High Shear Mixer	5, 10, 30	140–145	20	5000
[87]	Mechanical	2, 4, 6, 8, 10	150	30	1000
[53]	High Shear Mixer	1, 2, 3		10	5000
[5]		5	135	30	1800
[88]	Mechanical Mixer		110	15	2000
[89]	High Shear Mixer		150	30	900
[90]	High Shear Mixer		170	50	4000
[91]	High Shear Mixer		120	120	900
[92]	High Shear Mixer	0, 2, 4, 6, 8, 10	200		3000
[93]	High Shear Mixer	0, 4, 8, 12, 16	160	40	5000
[94]	Stirrer	5, 10, 15, 20	135	15	2000
[95]	High Shear Mixer	1, 2, 3	163	60	1000
[96]	High Shear Mixer	5, 10, 15	140	30	4000
[97]	High Shear Mixer	5, 10, 15	140	30	4000
[77]	High Shear Mixer	0, 3, 6, 9	180, 200, 220, 240	120, 240, 360, 480	1500
[70]	Propeller Mixer	6	130	15	1200
[98]	Shear Device	2.5, 5.5, 8, 10.4	135	60	5000
[62]	Low-Shear Drill Mixer	1, 3	135	40	
[54]	High Shear Mixer	5, 10	140	60	1000
[66]	Glass Rod	5, 10, 15	150	5	Simultaneous stirring
[99]	Shear Mixer	20, 40, 60	120	60	1000
[100,101]	Silverson Shear Mill	0.75	140	60	3000
[102]	Silverson Shear Mill	0.5	140	60	3000
[17]	Hand Blended	10	135	5	
[103,104]	Mixer	5, 10, 15	150	10	500
[73]	Laboratory Mixer	10	160	10	500
[4]	Shear Mixer	10	135	30	750
[105]	High-Speed Shear Mixer	10, 15, 20	135	15	5000
[63,106]	Hand Stirring	1.25, 2.5, 3.75, 5.0	150	1	
[107]	Mixing	0, 9, 18	135		
[108,109]	Mixing	0, 6, 12, 18	140		
[110]	High-Shear Mixer	2:1, 1:1, 1:2	180	30	3000
[111]	Mechanical Stirrer	0–30	120	20	1500

## 4. Performance Evaluation

This section presents the key results and findings related to the performance and behavior of bio-asphalt binders and mixtures. Five aspects of performances were covered: (1) rheological properties, (2) high-temperature performance, (3) intermediate-temperature performance, (4) low-temperature performance, and (5) performance in asphalt mixtures. The properties of bio-modified asphalt binder were assessed through multiple laboratory tests such as the rotational viscosity (RV) test, dynamic shear rheometer (DSR), bending beam rheometer (BBR), etc., in addition to the rolling thin film oven (RTFO) and pressure aging vessel (PAV) tests to simulate short- and long-term aging conditions, respectively. The behavior covered low-temperature, intermediate-temperature, and high-temperature performances of these materials. Table 4 shows common testing practices with corresponding aging levels and temperature ranges.

### 4.1. Mechanical Properties of Asphalt Binder

The mechanical properties of BMB blends were investigated in numerous studies regarding density, penetration, softening point, rotational viscosity, ductility, storage stability, etc. These parameters represent the consistency and workability of the asphalt binder. The following sections discuss these different parameters.

#### 4.1.1. Specific Gravity and Density of BB

BBs extracted using thermochemical conversion techniques were higher in density and specific gravity than the asphalt binder in some cases [125,126]. Higher contents of BB, i.e., 100%, have a specific gravity of 1.33, compared to 1.04 in a conventional binder. Higher contents of the BB resulted in increased specific gravity, i.e., adding 50% of bio binder resulted in an increase of 12.5% and 10.38% compared to the control binder and 8% modified binder, respectively [125]. The density of the BB generated through fast pyrolysis of sawdust and woodchip was 1.1 g/cm^3^, compared to 1.03 g/cm^3^ for the conventional asphalt binder [86,127,128,129,130]. Another study reported a specific gravity of 1.2 for the BB produced from wood through the pyrolysis technique [27,29,131]. BB produced through fast pyrolysis of household waste has a specific gravity of 1.09 [132].

On the other hand, bio-oils such as WCO and DSO were reported to have a lower density of around 0.896–0.96 g/cm^3^ [64,70,77,80,81,83,94,96,97,133,134,135]. The densities of the waste oil obtained from different facilities were similar. The specific gravity of the BMB blend was reduced with the addition of the waste oil by 5.4% when 8% of the bio-oil was added [80]. The density of WEO was reported as 0.93 [136]. The specific gravity of bio-oil as a coproduct of manufacturing chemical alcohols from corn is 1.013, while the bio-oil as a residue of castor oil after refining fatty acids from castor is 0.987, compared to 1.03 for the conventional asphalt binder [1,84]. Examples of specific gravities of different types of BB/bio-oils from different studies are shown in Table 5. The change in the physical form of the BMBs was attributed to the changing consistency of the bio-oil.

#### 4.1.2. Physical Characteristics of BMB

The workability of the asphalt binder is measured by various testing methods, including penetration, softening point, rotational viscosity (RV), temperature susceptibility, ductility, etc. The low content of bio-oil is capable of changing the workability of the BMB blends as it improves the mixing of components in asphalt [62,126,138]. The effect of incorporating 2, 5, and 10% of swine waste bio-oil was investigated in a study [25,52]. The RV of the 5% modified asphalt binder was reduced by approximately 35–55% at 135 °C for the unaged conditions [25,52,139].

In another study, three percentages of bio-oil produced from wood were used: 5, 10, and 15% by weight, to generate bio-modified asphalt binder. Adding 5% of bio-oil resulted in a change of +38% and −5.4% in the penetration and the softening point, respectively. As a result, the penetration grade of the modified binder was anticipated to be increased from 50/70% for the base binder to 70/100% for the 5% BMB. Also, the rotational viscosity of the binder decreased by 35% compared to the base binder, thus reducing the mixing and compaction temperatures. However, the temperature susceptibility was not changed or affected [103,104]. The viscosity of the BB was found to be higher when the oil content was used as an extender. However, both blends showed Newtonian or near-Newtonian behavior at all testing temperatures and shear rates [125]. It was observed that the BMB was less susceptible to temperature changes. 

The addition of bio-oil derived from WCO and waste vegetable oil (WVO) derived from sources such as sunflower and rapeseed resulted in a decrease in softening point and viscosity and an increase in penetration and ductility for the control asphalt binder [33,37,87,140,141]. In an experimental study investigating the performance of asphalt binder modified with soybean oil, both new and waste oils were used in concentrations of 1%, 2%, and 3% of the asphalt binder. Adding oil resulted in an increased penetration index (PI) and a decreased softening point. Viscosity also decreased with the incorporation of oil into the binder. Minimal variation was observed when using waste oil [142]. In another study, the modified binder was softer than the conventional binder; therefore, the penetration was increased, and the softening point, as well as the viscosity, was decreased [95]. Flash and fire points were increased with the addition of oil. In addition, the workability was enhanced at lower working temperatures [80].

DSO and palm kernel oil (PKO-p) increased the penetration of the modified binders [64,88]. It was shown that the DSO-BMB were less susceptible to temperature changes, and the 1.5% DSO-BMB blend achieved the lowest VTS value among the modified blends. As a result, the mixing and compaction temperatures were reduced. The physical results of the DSO-BMB blends are shown in Table 6 [143]. PI and penetration viscosity number (PVN) for asphalt bitumen indicate lower temperature susceptibility; the results showed that the overall trend for PI and PVN increased subsequently with the increment of PU, which shows a reduction in susceptibility of the bitumen’s temperature. The recommended PKO-p and MDI content ratio to use was 1:0.6 with 5% of PU. However, it is suggested that a smaller percentage of PU, between 2–10%, be selected. A further study assessed the use of very high oil contents of binders (up to 60%) with a 30% weight of PKO-p of MDI [88,99]. The mixing and compaction temperatures were found to be decreased by 10% compared to the base binder (80/100 penetration grade). The performance results of bio-binders produced from palm oil empty fruit bunches in a study showed that temperature susceptibility was affected [54]. The penetration index was found to be decreased significantly (180%) when increasing the oil content from 5% to 10%, and the softening point was decreased by 35% as well. However, the modified binders’ viscosity was similar at oil levels and temperatures.

A study investigated the performance of two bio-asphalts made from corn (DC) and castor (SH) [1,84]. The maximum bio-asphalt content was determined to be 10% for DC and 15% for SH. This maximum content was selected as the optimal amount for replacing the base asphalt, meeting the requirements of specification JTG F40-2004. The VTS results indicated that bio-asphalt could increase the temperature susceptibility of the asphalt binder, while the penetration index results demonstrated that all three binders were classified as sol-gel types, i.e., the penetration index is between −2 and 2, and this classification reflects the colloidal nature of asphalt [83]. Physical properties of penetration, softening point, and ductility of bio-binder (BB), petroleum-based asphalt (BA), and 10% and 15% bio-based asphalt (BBA) indicated that BBA is softer than BA, as indicated by the low penetration and ductility. As a result, it is easy to assume that the viscosity of the 10% BBA and 15% BBA are higher than that of BA [84].

Different grades of asphalt binders were tested in a study to investigate the effect of adding rapeseed oil as a fluxing agent to asphalt binder [106]. The output of the study indicated that the degree of fluxing behavior is related to the bio-additive content. Even a small amount of bio-additive (below 2.5%) significantly lowers the binder consistency. Adding 5% bio-additive to the binder results in a softening point temperature drop of 13 °C to 15 °C. The suitability of asphalt binder containing castor oil-based bio-asphalt for paving applications was evaluated using conventional laboratory tests [111]. The penetration increases considerably with increasing bio-asphalt content at a given temperature, indicating a softening effect of the bio-asphalt. This indicates that penetration values at lower temperatures (5 °C) are generally lower than those at higher temperatures (15 °C and 25 °C). This lower penetration at colder temperatures is expected because materials typically become harder and less penetrable as temperatures decrease. However, the rate at which penetration increases with the addition of bio-asphalt is more pronounced at lower temperatures. This suggests that bio-asphalt significantly reduces the temperature susceptibility of the binder, making it softer relative to its behavior at these temperatures. Asphalt binders of penetrations 50, 70, and 90 are obtained by adding 3.0%, 12.7%, and 21.8% bio-asphalt, respectively. The softening point decreases slightly with increasing bio-asphalt content; however, the ductility remains sufficient when the bio-asphalt content is below 15%. At 15 °C, ductility significantly decreases from approximately 100 cm to 44 cm as the bio-asphalt content increases from 15% to 30%.

The diffusion mechanism of the bio-modified asphalt binder was studied [98]. The viscosity of the binder was found to be reduced dramatically by the addition of 2.8% of the bio-binder; also, increasing the temperature from 298 K to 333 K lowered the viscosity by 90%. The ductility of BMA decreases with the increase in temperature, but the ductility increases as the bio-oil content increases. The properties of asphalt binder modified by bio-oil derived from waste cooking oil were studied [33]. The consistency of the material was tested using penetration, softening point, ductility, and RV tests. The addition of bio-oil resulted in a decrease in softening point and viscosity, as well as an increase in penetration and ductility for the control asphalt binder. Another study evaluated the mechanical performance of the modified binder by waste cooking oil [37]. A decrease in viscosity was obtained with the introduction of bio-oil. The mixing and compaction temperatures were reduced with the addition of bio-oil. WVO is used in sunflower and rapeseed oils. The use of oil increased penetration and decreased the softening point [140,141].

An experimental study on using WVO as a modifier in the asphalt in the asphalt binder was performed [80]. Various oil contents between 0 and 8% by volume of asphalt binder were used. The addition of oil increased the penetration of the binder due to the change in the consistency of the modified binder. The ductility of the modified binder improved while specific gravity was reduced with the addition of oil. Adding oil also increased the softening point and temperature susceptibility of the binder. Flash and fire points rose with the addition of oil. Furthermore, the RV decreased, enhancing workability at lower working temperatures. A review that assessed the modification of asphalt binder by bio-oil generated from different biomass sources stated that the bio-oil would be capable of changing the rheological properties of the asphalt binder, increasing the viscosity with higher oil contents and service temperatures [126].

#### 4.1.3. Storage Stability

The thermal storage stability of asphalt binders is measured in terms of the separation or segregation tendency of the bio-oil or BB in the BMB blend. BMB blends produced in a study showed no significant variation in the rutting parameter (|G*|/sinδ) between the top and bottom portions of the blend up to BB content of 25%. However, in higher content, i.e., 50% BB, a statically significant difference was observed, as in Table 7 [125].

The thermal storage stability of bio asphalts decreased with increasing bio-oil content and storage temperature [33,144]. The differences in the softening point between the modified binder’s top and bottom (ΔTR&B) can be less than 2.5 °C for the different oil contents. When bio-oil content was 10% at a storage temperature of 120 °C, ΔTR&B was 1.4 °C, whereas it could reach as high as 9.2 °C when the bio-oil content was 30% at a storage temperature of 180 °C. This indicates that bio-oil is compatible with asphalt. Different types of bio-oil/BB resulted in a decrease in the separation index of surface-activated rubber asphalt blends [145]. Incorporating bio-oil generated from wood pallets reduced the separation index of surface-activated rubbers asphalt blends by 80%, followed by WVO. 

### 4.2. Rheological Properties of Asphalt Binder

The characterization of the rheological properties of the BMB blends includes testing asphalt blends at different temperature ranges and aging conditions. The following sections provide an extensive discussion of the results in the studied literature in terms of frequency and temperature sweep, rutting, and fatigue resistance performances. The DSR tests showed that the modified binder is more susceptible to heat changes. These results can be concluded in terms of rutting and fatigue resistance capability, where the modified binders possess less resistance to rutting but good resistance against fatigue [91].

#### 4.2.1. High-Temperature Binder Performance

The high-temperature performance of asphalt binder is characterized by testing asphalt at a temperature of 40–70 °C at multiple frequencies, as per testing standards, to assess performance parameters such as resistance to rutting and cracking. The DSR frequency sweep test was carried out to study the binder behavior within the linear range using the parallel plates geometry mainly following the ASTM D7175 at a high-temperature range to determine the dynamic shear modulus (G*) and phase angle (δ) values of BMB blends using a wide range of oscillatory shear loading frequencies [103,116,146]. The DSR results showed a reduction in |G*| values for the BMB blends compared with the base binder. Swine manure binders also showed lower modulus values compared to standard binders. This drop is expected to reduce the rutting resistance parameter (|G*| /sinδ) of the asphalt blend. Major reductions were observed due to the addition of the bio-binder [51,52,139]. 

This indicates that bio-oil has an unfavorable contribution to the high-temperature stable property of asphalt [98]. However, at low oil contents, i.e., 1 and 2%, G*/sinδ was found to be almost equivalent to that of the nonmodified binder [86,95]. The influence of adding WCO to different asphalt base binders was investigated. It was concluded that the rheological properties were changed, demonstrated by the decrease in G* and increase in the phase angle (δ) parameter with the increase in WCO [6,7,33]. Further research was conducted that studied the effect of adding soybean oil to the asphalt binder [147]. It was concluded that G* decreased with the increase in oil content due to the plasticity effect.

A used frying oil (UFO) was obtained in a study [92] from commercial restaurants. The study also assessed using the TLA to replace the conventional Trinidad petroleum asphalt binder (TPB). It was found that the complex modulus (stiffness) of the TLA was higher than that of the TPB, and the addition of the UFO resulted in significantly reducing the complex modulus by 85 to 90% when introducing 2% of UFO to both TLA and TPB. The phase angle was increased when adding the UFO, which reduced the modified binder’s elasticity. The TPB phase angle was higher by 28% compared to the TLA binder. However, the addition of 2% UFO did not affect the phase angle of both binders, but when adding 4%, a 12% increase was obtained for the TLA binder compared to a 2.25% reduction in the TPB binder. The phase angle increased to 6% with the addition of UFO to the TLA binder, while the trend was not stable for the UFO contents in the TPB binder. TLA and UFO-TLA modified blends showed significantly lower values of δ and higher values of G*, confirming the superiority of the TLA material.

The vegetable oil was compatible with asphalt binder, so the designer could modify the conventional asphalt at any desired viscosity or G* value [148,149]. A 6% vegetable oil was blended with asphalt binder Pen 10/20 to produce a Pen 40/60 asphalt binder named Vegetex 50. The sweep test results showed that G* and δ values were almost identical at all aging levels for the virgin Pen 40/60 and Vegetex 50 asphalt binders. The master curves were found to decrease monotonically when increasing the bio-oil weights. The unrecovered strain during the MSCR test was found to be increasing at higher temperatures and oil contents [97,150].

According to the results of the rutting parameter, the high content of pyrolyzed empty fruit bunch (EFB) bio-oil can improve the rutting resistance of bitumen at high temperatures and give a comparable result to virgin bitumen [54]. When the temperature was below 60 °C, it caused a decrease in the complex stiffness modulus by 92%, i.e., 2.5 × 10^7^ Pa to 2.0 × 10^6^ Pa [106]. The hardening of the binder with 2.5% bio-additive added is comparable with the non-fluxed binder. The tested bio-additive demonstrates higher effectiveness at concentrations of 3.75–5.0%. Softer binders are less prone to hardening than harder ones. When a fluxing agent is added to road bitumen, it increases the viscous component of the bitumen’s complex modulus. This effect is similar to what happens when the temperature of the bitumen is raised [63]. These results highlight the impact of bio-oil on enhancing the flexibility and reducing the stiffness of asphalt binders, particularly at high temperatures.

It was determined that the rutting parameter was reduced with the increase in the bio-oil content. However, the rutting resistance was better for the modified binder with a DSO content of less than 2.5%. although the PG dropped from 64 to 58 °C. The traffic level of the 2.5% BMB was found to be enhanced to heavy instead of standard for base binders but with higher results of unrecoverable strain values [64]. The addition of DSO bio-asphalt enhanced rutting resistance, resulting in higher dynamic stability and reduced rutting depth, in contrast to SH bio-asphalt [1].

The low phase angle indicates better elastic recovery of the asphalt binder without oil; thus, adding oil reduces elastic recovery. The decrease in G* compared to the pure binder diminished resistance to deformation before and after aging. Both new and used oils in binder modification produced similar results [147]. The incorporation of the UFO in the blends led to a decrease in the rutting resistance [92].

#### 4.2.2. Intermediate Temperature Binder Performance

The fatigue life of asphalt pavements occurs at intermediate service temperatures, e.g., 10–30 °C. These viscous components in the BMB increase the fatigue performance of asphalt [151,152]. Higher percentages of bio-oil in modification improved the resistance to fatigue cracking due to the reduction in the fatigue parameter (G*sinδ) [103,143,153]. Superpave limits the G*sinδ to be less than 5000 kPa. This criterion was satisfied at 18.3, 17.0, 15.2, and 14.2 °C for the control, and 1, 3, and 5% binders, respectively [153]. Bio-oil effectively reduced the |G*|sinδ values of the binders, indicating improved fatigue resistance. Higher concentrations of each bio-agent lead to more significant reductions in |G*|sinδ. This improvement in fatigue performance is essential for extending the lifespan and durability of pavements subjected to moderate climatic conditions [153,154]

The LAS test indicates that the fatigue life was enhanced when bio-oil was added, expressed in terms of the fatigue failure cycles (N_f_) [146,155]. At certain stress levels, N_f_ was found to be increased as per the visco-elastic continuous damage (VECD) theory [64,143]. The shear stresses and strains obtained from the raw results of the LAS test at the intermediate temperatures and 20 °C as comparison temperature indicated that the effective shear stress for modified binders was reduced by adding the DSO to the binder. This result was attributed to the energy required for the modified binder to resist fatigue cracking. The maximum effective shear strain at the intermediate temperature was in the range of 10 to 15% [143]. The analysis of damage trends reveals that increasing the bio-oil content generally enhances the material’s integrity and resistance to fatigue damage. Higher percentages of bio-oil, such as 4.5% and 5.5%, show similar performance levels, indicating that beyond a certain concentration, additional bio-oil does not significantly improve fatigue resistance. At lower temperatures, the reduction in the C parameter is more pronounced, highlighting the significant impact of colder conditions on fatigue resistance. As the temperature approaches intermediate levels, the fatigue resistance of the binders becomes less sensitive to the exact bio-agent content, suggesting a convergence in performance across different bio-agent concentrations.

#### 4.2.3. Low-Temperature Performance

Cold climate regions often experience non-load associated cracking, or what is called thermal cracking, when thermal stresses build up during cooling events in the pavement and exceed the tensile strength of the asphalt concrete layer [156]. BB has extensively become a research alternative to the conventional asphalt binder and has proved to be effective in decreasing long-term aging-related distresses [152,157]. The thermal cracking resistance of asphalt binder is assessed through testing at low temperatures, including the BBR test protocol. The increased ductility of modified asphalt leads to improvement in their thermal and low temperature cracking resistance and the stress relaxation property [33,155].

The BBR results showed lower creep stiffness and higher m-value and induced jump in the lower temperature grade, which indicates an improvement in the thermal cracking performance of the bio-binder compared to the conventional binder. The cracking temperature was found to be decreasing with the addition of bio-binder [25,95,158]. The BBR results showed that the cracking temperature was reduced to −24 °C compared to −12 °C in the 5% modified binder compared to the control binder as both m-value and stiffness passed the Superpave^TM^ requirements. The m-value of the modified binder was reduced by 5.2% at −12 °C [139]. The lower testing temperature showed an increase in the stiffness of the bio-modified binder, as shown in Table 8 The BBR results showed a reduction in the creep stiffness and increased m-value of the BMB blends [33,159]. The stiffness of the modified blends was reduced by 65% when the bio-oil content was increased to 9% instead of 3% [159].

Oils, including bio-based and refined waste oil, were used to investigate the low-temperature performance of asphalt [155]. The study found that the bio-oil reduced the stiffness of the modified binder by 60%; however, the m-value was increased. It can be observed that the oil can significantly lower the continuous grading temperature up to 9 °C and lower glass transition temperatures, indicating a substantial improvement in low-temperature environments. 

The shear modulus and the bending creep stiffness were found to be decreased. The m-value results were found to be increasing, which indicated an enhancement in the thermal cracking resistance for asphalt binder [33]. The addition of 5.5% DSO resulted in decreasing the low-temperature grade to −28 °C compared to −16 for the unmodified asphalt. On the other hand, a lower content of DSO, 1.5%, was optimum in terms of painting high-temperature performance at 64 °C and extending lower temperature grade to −22 °C. Further addition of DSO reduced both low and high temperatures PG of the BMB. From BBR results, it was determined that the asphalt binder was able to achieve the Superpave specs at −18 °C in the 5.5% BMB, which was increased from −6 °C for the control binder [64,143].

The results of the BBR test showed that the stiffness of BBA is higher than that of BA, except for 10% BBA at −24 °C. Similarly, the m-value of BBA is slightly lower than that of BA, except for 15% BBA at −18 °C. This indicates that asphalt binders become more prone to cracking at lower temperatures [84]. Additionally, compared to 10% BBA and 15% BBA, an optimized content of BB can be determined, resulting in a BBA with a relatively lower stiffness and higher m-value.

#### 4.2.4. Aging Resistance

Oxidative aging is the main mechanism of asphalt aging, where oxidation reactions take place for some fractions of asphalt binder [160]. The use of aging index ratio, the ratio of aged to unaged performance parameters such as penetration, softening point, and viscosity, to assess the aging resistance of the BMB in terms of viscosity, softening point, penetration, or rutting parameter (G*/sinδ) was stated in different studies. The aging index for different BMB blends showed a higher index than the conventional binder by 17.64 and 18.82% for the BMB blends containing 5 and 10% BB, respectively [27,87,161]. Table 9 shows the aging index for BMB containing 25% TLA and varying contents of bio-oil in terms of penetration, softening point, and rotational viscosity in a study [162,163,164]. The BMB blends were more susceptible to aging, as expressed by the increase in the aging index [87,142,161,162].

The thermo-oxidative aging of the unaged BMB is mainly attributed to the volatilization of light components of the BB, i.e., fatty acids and esters [10,165]. The BMB is considered to become stiffer after aging as the mass loss increases at higher oil contents [55,95,129]. In another study, it was found that the mass loss due to RTFO-aging of bio-asphalt increased at higher oil content [86,166]. The mass loss after RTFO was lower than 1% and considered as an acceptable value for paving applications [103,140,141]. Characterization of rheological properties and aging mechanisms with a high percentage of bio-binders (30 and 70% by weight) was conducted [166]. The swine waste BB resulted in a decrease of about 29% in the rolling thin film oven (RTFO)-aged BB viscosity compared to the RTFO-aged base binder, and the pressure aging vessel (PAV)-aged binder experienced a viscosity reduction of about 63% when compared with the PAV-aged base binder [139]. Bio-asphalt can significantly improve the aging resistance of the SBS-modified bio-asphalt binders, although the improvement effect decreases as the bio-asphalt content increases [130].

A temperature sweep test of the Buton rock asphalt (BRA) BMB blends was carried out at different aging levels in a study [167]. The 5% modified blends without BRA content tended to become stiffer with the longer aging time. G*/sinδ of the 5% bio-oil blend, 20 h of PAV, was less than the control asphalt by 16%. The reason is that adding bio-oil increases the proportion of the light components of the BRA-modified bio-asphalt, and it is more volatile and oxidized during the pressure aging process. On the other hand, because rock asphalt belongs to hard material, adding rock asphalt will harden the modified asphalt and increase its modulus. As a result, the rutting index is significantly increased, even exceeding the neat asphalt, which is consistent with the expected effect of rock asphalt. 

### 4.3. Effect of Adding Bio-Oil as Rejuvenating Agent

The use of asphalt mixtures containing reclaimed asphalt pavement (RAP) from milled pavements has been practiced for over half a century. Asphalt aging begins as soon as the mixture is produced, with oxygen reacting with the polar components of asphalt, purportedly converting polar aromatics and resins to asphaltenes. Bio-oil is a promising rejuvenating agent for aged asphalt binders, and it has been shown to increase the viscous component content of aged asphalt due to its high viscous component content. This suggests that bio-oil can effectively rejuvenate aged asphalt. Adding bio-oil decreases the softening point and viscosity while increasing the penetration and ductility of the control asphalt binder. Separation tendency tests indicate good bio-oil and control asphalt compatibility under static heated storage conditions. A new Food Waste Bio-Oil (FWBO) rejuvenator has been proposed and investigated for its effects on aged asphalt binder, artificial RAP (ARAP), and RAP’s rheological and cracking performance, compared to two different market rejuvenators [168,169,170,171].

Many research studies have accepted the use of RAP and RAS in asphalt pavement to enhance the sustainability of the asphalt binder in terms of cost savings and environmental footprint of materials used [172,173,174,175,176]. The aged asphalt has higher stiffness measurements for lower penetration and ductility values than the fresh binder. Multiple techniques are used to reduce the stiffness of the aged asphalt binder, such as the incorporation of different additives such as the warm mix additives (WMA), the use of softeners such as bio-oil, or a combination of both [177,178,179]. Bio-oil or recycling agents (RAs) are formulated to rejuvenate or soften aged asphalt binder, RAP, and RAS materials, thereby restoring the properties of fresh asphalt binder. These agents are effective when the oil content successfully reinstates the original characteristics of the asphalt binder. Based on viscosity results, it has been demonstrated that a 4.5% dosage of rejuvenator is sufficient to restore the viscosity of the 70/100 asphalt binder. This indicates that RAs can significantly improve the performance of aged binders, enhancing their workability and extending the lifespan of asphalt pavements [22].

A bottom-up study evaluated the moisture susceptibility of bio-modified binders and HMA-containing RAP [180]. Adding 5% and 10% of BMB to the conventional asphalt binder (PG64-22) through cohesive moisture susceptibility was tested for the BMB binder using the MSCR test for samples conditioned in water at 25 °C for 120 h. It was shown that adding the bio-binder to the virgin asphalt binder increased the nonrecoverable creep compliance (J_nr_) by 17%and 122% with the addition of 5% and 10% BMB, respectively. On the other hand, the J_nr_ value was lowered due to the moisture conditioning by 28% in the control binder, while the 5% and 10% were lowered by 14% and 26% compared to their unconditioned state, which in return indicates an increase in the recoverable creep compliance within asphalt binder. The direct adhesion test was conducted to evaluate the effect of the moisture on the binder. The testing samples were prepared as per AASHTO TP89; the samples were then placed in a temperature-controlled water bath for 120 h at 6 °C to ensure complete adhesive failure due to the brittleness of the binder. 

The storage and loss modulus of the BMB were obtained at 25 °C at different frequencies. Both moduli were increased at higher frequencies; manure-based BMB had the highest storage modulus, followed by algae-based BMB, and finally the co-liquified rejuvenator (50% swine manure + 50% algae). The delta crossover modulus and frequency values, defined as the difference of crossover modulus between pure aged and rejuvenated samples, showed positive values. This suggests that all the bio-rejuvenators effectively increased the crossover modulus and frequency values [17,181]. 

Furthermore, other experimental studies aimed to investigate the performance of bio-modified RAS (BMS) by introducing 10% of swine manure bio-binder to different contents of RAS [4]. As a result, the mixing temperature was reduced from 180 °C to 135 °C. The degree of blending between the RAP/RAS and the bio-modified binder was relatively good [182]. The effect of adding RAS to the neat asphalt was eliminated by adding the bio-binder, as explained by the viscosity test results. As the percentage increases to 30% and 40%, the viscosity further decreases, demonstrating that higher concentrations of RAS and BMS continue to enhance the binder’s workability at elevated temperatures. This trend suggests that modifying asphalt binders with appropriate dosages of RAS and BMS can effectively lower viscosity, thereby improving the handling and application of asphalt mixtures.

Hence, an increased shear susceptibility was obtained due to the deviation of the Newtonian behavior of the fluid alleviated by the addition of BMS. As the RAS content increases, so does the complex modulus (G*), where a significant drop was obtained at high frequencies, which indicates the enhancement of rutting resistance at slow traffic. The RAP bio-modified asphalt binder at similar dosages also showed similar behavior in [182] compared to the results of [4]. The Superpave specification volumetric targets were achieved at 40% RAP and 5% of BMB. The fatigue, rutting, and moisture susceptibility introduced by the RAP binder were eliminated in asphalt mixtures where similar performance to the control mixture was obtained. 

The fatigue resistance properties of the bio-rejuvenated asphalt binder increased with higher concentrations of the bio-rejuvenator. It was concluded that a 15% concentration of bio-oil is recommended for rejuvenating aged asphalt binder for reuse in pavement construction. The G*/sinδ for BR-15% at 10 Hz decreased by 71.7%, 73.1%, 74.8%, 75.7%, and 76.4% compared to the aged asphalt at temperatures of 52 °C, 58 °C, 64 °C, 70 °C, and 76 °C, respectively [105].

An asphalt binder with PG58-28 was selected as a base binder in a study [105]. A 10% bio-oil was used as a rejuvenator for aged binders (BR10%). The rotational viscosity was reduced by 21.9% at a temperature of 135 °C. This indicates that adding bio-oil enhances the viscous components and can rejuvenate the viscoelastic properties of aged asphalt binders to near-original levels. Samples with bio-asphalt derived from wood waste exhibited greater fatigue life and better storage stability compared to those with bio-asphalt derived from corn [183,184].

On the other hand, the addition of bio-oil to the RAP binder lowered its softening point, viscosity, and high PG [87]. The Superpave high-temperature PG of the RAP binder with 10% bio-oil was PG70, which is similar to the base binder. Therefore, a 10% oil content was identified as the optimum dosage for incorporating bio-oil with RAP binder as a rejuvenator. Further research assessed the effectiveness of bio-binder (A, which is composed of 10% bio-oil with RAP binder) [73]. The results showed that adding the bio-binder to the control binder increased the penetration more than twice that of the base binder. Furthermore, the recycled blends containing bio-binder were less susceptible to aging than the conventional binder blends. The results indicated that all the recycled blends had identical curves at all aging levels compared to the base binders. The fatigue behavior of the binder was assessed through the fatigue parameter, |G*|sin(δ); it can be observed that the fresh binder A had a low value compared to that of the RAP binder and the bio-RAP. The bio-RAP reduced fatigue parameters by almost 50% compared to the RAP binder, and the A + bio-RAP was 70% less than the bio-RAP. The nonrecoverable creep compliance (j_nr_) at 70 °C was increased with the different binders due to the reduced viscosity of the modified binder, which in turn decreased the j_nr_ and the recovery % of the binders. The hot recycling of reclaimed bio-asphalt (bio-RAP) reduced the susceptibility of the mixtures to cracking compared to the recycling of conventional reclaimed asphalt (RA). Additionally, using bio-binders in the hot recycling of conventional RA may be beneficial for reducing cracking due to the lower aging rate of the binder. However, the reduced aging experienced by the blends with the bio-binder resulted in a higher tendency for rutting in the asphalt mixture.

In another study, modified PAV-aged asphalt binders PG58-28 and PAV PG64-22 were evaluated [170]. The rheological properties of bio-rejuvenated asphalt binders were evaluated using RV, DSR, and Asphalt Binder Cracking Device (ABCD) tests. It was found that as the bio-oil content increases, the flow activation energy of bio-rejuvenated asphalt decreases. Compared to virgin and aged asphalt, bio-rejuvenated asphalt can endure higher thermal stress caused by cooling. Bio-oil can restore the low-temperature crack resistance of aged asphalts PAV PG58-28 and PAV PG64-22 to the level of virgin asphalt or even better. The performance grade of aged asphalt PAV PG58-28 (PG90-25) can be restored to PG77-30 with 15% bio-rejuvenator, and the performance grade of aged asphalt PAV PG64-22 (PG92-21) can be restored to PG78-24 with 20% bio-rejuvenator.

Further, the RAP was blended with virgin asphalt binder and bio-oil as a rejuvenator, waste cooking oil, and cottonseed oil [35]. The rutting resistance factor, phase angle, complex modulus, failure temperature, and rotational viscosity were determined. One study examined the effect of adding WVO to aged asphalt binders with varying oil contents [83]. The amount of oil penetration increased as the amount of waste vegetable oil increased. Oil content increased the rutting resistance factor as the viscosity and softening point decreased. In contrast, ductility increased as the oil content increased. Excessive amounts of waste oil reduced elastic recovery performance. The complex modulus decreased as oil content increased, while maximum and permanent deformation increased with higher waste oil dosage. When aged bitumen was blended with 0–5% waste cooking oil, the penetration value increased with higher oil content, as the asphaltenes-to-maltenes ratio decreased [36]. At 3% oil, penetration and softening points resembled the original bitumen values. With a higher oil content, viscosity values and dynamic shear modulus decreased. The phase angle increased with increasing waste cooking oil content. The optimum oil content was around 3–4%, which achieved good physical and rheological properties compared to the original binder.

A study assessed the use of waste cooking oil in the modification of asphalt binder and mixtures [185]. Two types of bio-based additives were used, WCO and factory-produced, and both were mixed with RAP binder. The rheology of the blended binders, including the complex modulus and viscosity, was found. It can be noted that the addition of the bio-oil significantly reduced the complex modulus of the recovered asphalt binder. In addition, the viscosity of the modified binder was decreased with the increase of oil content and temperature. The amount of the new clusters in the asphalt mixtures was determined depending on the RAP content, aggregate petrography, RAP sources, additive type, RAP, and aggregate grain size. It was found that the number of new clusters formed in the mixtures with bio-additives was slightly higher than that of the control mixture, probably due to the softer asphalt binder. However, both additives increased the number of RAP clusters in all fractions. This means that additives made the bitumen softer, ensuring a stronger bond between RAP particles.

The implementation of waste cooking oil as a RAP rejuvenator was studied [76,186]. Waste cooking oil was found to be a suitable rejuvenating agent for aged asphalt binders. It was determined that the oil content is a statistically significant parameter that led to changes in the rheology of the rejuvenated asphalt binder. The penetration and the phase angle were increased regardless of the asphalt binder used in the study. However, adding bio-oil significantly affected properties such as softening point, viscosity, and complex modulus. The analysis of variance (ANOVA) test was conducted to determine the significance of the oil content on the different parameters. Adding 1% of the oil to the rejuvenated asphalt binder showed a statistically significant change in the penetration value. The use of rejuvenators, including waste vegetable oil, grease, and organic oil, was assessed. Five rejuvenators were added to be effective in maintaining or increasing low-temperature creep compliance and, at the same time, increasing the indirect tensile strength and fracture energy, improving the mixtures’ low-temperature performance. Four of these rejuvenators also reduced the penetration of the RAP binder to the required level of the virgin mixture. The high PG for all rejuvenated blends remained higher than that of virgin binders, indicating increased rutting resistance. High PG temperature correlated well with the Hamburg Wheel Tracker Test, proving that all recycled mixtures have high rutting resistance. Fatigue resistance of recycled mixtures at the used test parameters was higher than that of virgin mixture for all except the WEO rejuvenated mixture [107,108,109].

The feasibility of using bio-oil originating from soybean oil as a rejuvenator for aged asphalt was assessed in a study [81,187,188]. The addition of the waste oil to the aged binder resulted in a reduction in the viscosity. It was determined that 20% of waste oil resembled the original viscosity of the fresh binder; in return, the mixing and compaction temperatures were reduced. Adding 1% waste oil can reduce mixing and compaction temperatures of aged asphalt by 1.25 °C and 1.33 °C [81]. A bio-oil dosage of 2 and 3% can resemble the original softening point of the fresh binder [188]. Furthermore, the rheological characteristics can be enhanced at certain bio-oil dosage ranges at low and high-temperature ranges. Bio-oil is expected to increase the antiaging properties of BMB blends when the BA content is less than 4% [187].

Neat asphalt exhibits the highest rutting index, indicating greater stiffness, while bio-modified asphalt shows lower indices, with higher bio-oil content leading to increased flexibility. As aging progresses from 5 to 25 h in PAV-aged asphalt, the rutting index decreases for all samples. Neat asphalt remains stiffer, whereas bio-modified asphalts become more flexible, with longer aging durations amplifying these differences. The addition of bio-oils consistently lowers the rutting index, enhancing flexibility and reducing stiffness, especially at higher percentages. Overall, bio-oils improve the flexibility and rutting resistance of asphalt binders, thereby enhancing long-term pavement durability [189]. As bio-oil content increases, the G*/sinδ of asphalt decreases because the proportion of asphalt components changes with the addition of bio-oil. Aging increases the G*/sinδ of asphalt, indicating that aged asphalt has better high-temperature stability and greater resistance to rutting damage. Despite increased bio-oil content, the low-temperature behavior of long-term aged asphalt can recover to a level superior to that of neat asphalt. The minimum bio-oil content required for asphalt with different aging degrees was determined based on its low-temperature performance. 

Adding waste oil in dosages higher than 5% increased the fatigue resistance exponentially. The flashpoint was measured as a safety property, and it was determined that the addition of the oil to the aged binder reduced the flashpoint [94]. As a result, adding the bio-oil was risky in terms of safety. The mass loss of the rejuvenated asphalt was increasing compared to the aged asphalt and the base binder. Physical tests, such as the penetration, softening point, and ductility, were performed on the rejuvenated asphalt. The penetration value and the ductility of the rejuvenated asphalt were found to be equivalent to the base binder (Pen70) when the oil content is around 10%, which indicates that the relationship decreases with the increase of the oil content. Adding 13% bio-oil achieved a softening point in the modified asphalt that was comparable to that of the original, unmodified asphalt.; the relationship was adversely proportional. From the rheological performance perspective, it was determined that as the dosage of the waste oil increased, the workability was increased, and the fatigue life and the low-temperature properties were enhanced. In addition, the optimum oil content to achieve the best high-temperature performance for the rejuvenated asphalt was calculated as 13.4%.

A study showed that the use of the bio-oil generated from waste cooking oil biodiesel can enhance the workability and rheological properties of aged asphalt [133]. The use of 1.75% bio-oil rejuvenated the aged asphalt binder to the virgin level; 2% bio-oil recovered aged SBS asphalt’s penetration and ductility toward its original level. The low-temperature cracking resistance characterized by stiffness and m-value was improved significantly. However, the rutting index was better for the rejuvenated binder than the base binder. The PG temperature was 70 °C compared to 64 °C for the unaged base binder. 

The results indicated that the penetration rate of the bio asphalt increased at the test temperature (25 °C) to 70 penetration units for the 6% modified asphalt compared to 24 in the 0% aged asphalt. This value was reduced by 22% when subject to RTFO aging. The softening point was reduced by 15% compared to the recovered asphalt binder. The calculated penetration index was found to be within the allowable range (−1 to +1). Temperature susceptibility was found to decrease as the oil content increased. The modified asphalt showed higher resistance to short-term aging, as presented by the penetration index, before and after aging [135]. Viscosity–temperature susceptibility (VTS) results showed that RTFO composites with bio-oil are more susceptible to viscosity changes with temperature [190]. It was found from rutting (G*/sinδ) and fatigue cracking resistance (G*sin(*δ*)) parameters that for the modified binder, the rutting resistance decreased as temperature and oil concentration increased. The critical temperature, determined from DSR and BBR through high to low temperatures, reduced from 60 °C to 46 °C and 30 °C when 5 and 10% bio-oil were added, respectively. This reduction was eliminated with the addition of polymers to the binder with lower oil content. 

To investigate the performance of RAP asphalt binder rejuvenated by DSO, multiple studies were conducted [69,191,192]. Bio-oil concentrations of 0–10% were added to varying amounts of RAP (0–30%). The DSO reduced the G*/sinδ rutting parameter. Higher oil content resulted in higher strain and lower recovery percentages associated with increased J_nr_ values. Binders containing DSO were observed to be less susceptible to fatigue failure, with the number of cycles to failure increasing as the oil content increased. The creep stiffness of the modified binder decreased with higher oil content and lower temperatures. It was found that using DSO as a rejuvenator helps restore the original characteristics of the asphalt binder. The addition of DSO improved the fatigue life of asphalt mixtures containing 15% to 20% RAP [69,191].

Another research used two different bio-oils, namely, Pongamia oil and a composite oil made of castor oil and coke oven gas condensate, which were used as rejuvenating materials for aged asphalt binder [66]. It can be concluded that the 5% oil rejuvenated aged binder showed desirable rutting and fatigue behaviors even better than the virgin binder. Though the fatigue performance of the rejuvenated binders with increasing oil percentage was much better, their rutting performances were poorer than the virgin binders (except for 5% oil-rejuvenated binders). The Pongamia oil was found to be better in the case of fatigue performance, while the composite oil was better in the case of rutting performance. It was found that the oils could be considered suitable rejuvenators for effectively restoring the properties of the aged binder. 

A study added a low oil content from soy fatty acids (SFA) to different binders [62]. The original binder and the binder at both modification levels demonstrated a linear relationship between viscosity (on a log scale) and temperature. The viscosity was lowered by increasing both the SFA content and the temperature. Through the performance grading limits of linearity, it was important to test if the modified binders perform linearly under an initial strain of 2% (the linearity limit is 10%). It was determined that adding SFA did not negatively affect the linearity. The SFA-modified binder with RAP binder behavior at service temperatures as compared to the RAP binder. RAP binders usually possess sufficient stiffness to resist permanent deformation at high temperatures. However, to ensure adequate workability and resistance to cracking, they require the addition of softer binder grades or recycling agents. Thus, adding the SFA to the RAP reduced the stiffness of the RAP binder and enhanced the workability of a lowered complex modulus at a given temperature. The bio-derived soybean rejuvenator additive was mixed with base binders PG64-28 and PG58-28 in a study [85]. The G* master of the modified binders was found to be reduced at both high and low temperatures. In addition, the aging characteristics were not influenced by the addition of the rejuvenator. The PG was reduced from PG58-28 to PG40-34, which means that the modified binder will be enhanced in terms of fatigue resistance and will be more susceptible to rutting than the unmodified binder. Such a significant change in the performance with this low dosage of bio-oil means that an even lower dosage can be designed to tailor the properties of the blend without greatly sacrificing the rutting resistance. A 6% and 12% oil content modified binders were blended with RAP binder to be used in the asphalt mixture [193]. The PG of the RAP-modified binder was reduced from 101-10 to 76-16 and 70-22 using the 6% and 12% oil content, respectively. However, an enhancement in the fatigue life of the binder was obtained according to the LAS results, whereas the intermediate temperature dropped from 31 °C to 25 °C. Higher fracture energy was obtained for the 12% soybean-modified PG58-28 asphalt mixture compared to the one with a neat PG58-28. 

Different rejuvenators, including extracted natural seed oil (referred to as “A”), cashew nut shell oil (referred to as “B”), and tall oil (referred to as “C”), were used to evaluate their effects on the aging of RAP (Reclaimed Asphalt Pavement) modified with rejuvenation agents, aiming to characterize how each rejuvenator influenced the properties of the aged RAP [194]. The study demonstrated that aging could significantly affect the rheological behavior of rejuvenated RAP binders. The differences between the unmodified and modified RAP binders were significant across the entire frequency spectrum. Specifically, RAP +5% B and RAP +5% C exhibited lower moduli than virgin bitumen 50/70. It was found that the RAP binder modified with 5% of rejuvenators B and C had softer mechanical properties than virgin bitumen 50/70, while the RAP binder with rejuvenator A was harder at low frequencies. Rejuvenators can soften the RAP binder across all frequencies. At lower frequencies, G″ (the viscous part of G*) was prominent for all materials, with RAP +5% C and RAP +5% B showing higher values of G″ compared to the other two rejuvenated binders, while virgin 50/70 and RAP exhibited similar G″ values. Conversely, at the highest frequency, RAP +5% B and RAP +5% C had higher phase angles, and thus higher G″ than RAP +5% A. All modified binders showed higher G″ than the virgin binder at high frequencies (low temperature), indicating a higher viscous component of the complex modulus than RAP, which is beneficial for low-temperature behavior. In terms of low-temperature performance, the rejuvenators had a positive effect on the unaged RAP binder: the fracture toughness temperature (TFTT) decreased after adding 5% rejuvenator, and this decrease was rejuvenator-dependent [195]. Rejuvenators could mechanically restore the aged RAP binder, showing significant differences in the modulus and phase angle results. Additionally, it was shown that the mechanical changes due to rejuvenators were not caused by changes at the chemical bond or functional group levels.

Adding 4% Jatropha Curcas oil to the aged binder effectively counteracted the stiffening caused by the 40/50 aged binder, resulting in properties similar to those of the 80/100 penetration binder. Adding a bio-based rejuvenator (JCO) to the partially aged binder reduced the stiffness of the binder (complex modulus) and its resistance to rutting and fatigue cracking, but it also increased the resistance to thermal cracking. Moreover, from the Aging Index Rutting Factor (AIRF) value, the binders containing a high amount of bio-based rejuvenator are susceptible to aging [196]. 

Researchers investigated the effect of adding waste engine oil to the asphalt binder containing an aged binder and virgin binder [197]. It was observed that adding waste engine oil decreased the viscosity, softening point, and complex modulus compared to the control binder. An increase in phase angle values was observed with higher oil content. A lower relaxation modulus was obtained with increased waste engine oil content. Reductions in viscosity and stiffness were recorded when 2.5% filtered waste engine oil was used as a rejuvenator for aged binder [198]. Low modulus values were recorded for the rejuvenated binders, indicating a possibility for better fatigue life.

### 4.4. Effect of Using Additives and Waste Materials in Combination with Bio-Oil

One of the challenges of using bio-oil to modify asphalt binders is the compatibility and stability issues caused by the different chemical compositions and polarities of bio-oil and petroleum asphalt [199]. To overcome these issues, various additives, and waste materials have been used in combination with bio-oil to enhance the interaction and homogeneity of bio-modified asphalt binders [2,199]. Some examples of these additives and waste materials are polymers, rubber, nano-particles, and plastic powder [52,57,129,200,201,202,203]. The following section states the effect of each additive type. 

#### 4.4.1. Polymers

Three different bio-oils, namely original (OB), dewatered (DWB), and polymer-modified (PMB), were derived from waste woods were used in asphalt binders and used to modify the PG58-28 asphalt binder by 5% and 10% [27,28]. It was determined that the viscosity of the modified binder was decreased by an average of 12% with the addition of 5% bio-oil. However, the PMB-modified asphalt binders were lower than both the OB and the DWB-modified asphalt binders. The aging index of the asphalt binders was increased with the increase of the bio-oil content by 17.64%, 18.82%, 18.15%, 26.13%, 6.62%, and 9.97% for the 5% and 10% OB, 5%, and 10% DWB, and 5% and 10% PMB modified asphalt binders, respectively, compared to the control binder. 

Adding polymers stiffened the modified asphalt binder, resulting in higher G*/sinδ than the other two oil types. After the RTFO aging test, the G*/sinδ was significantly higher than the control binder; the increase of bio-oil increased the G*/sinδ after aging. The bio-modified binder exhibits less temperature sensitivity than the control binder.

In a study, the high-temperature performance was evaluated using the DSR and MSCR tests [30]. The control asphalt binder used in this study was PG58-28; it was blended with 5% and 10% bio-oils (untreated bio-oil (UTB), treated bio-oil (TB), and modified bio-oil with polymers (PMB)) to prepare the bio-asphalt binders. The DSR test results showed that the addition of the bio-oils improved the high-temperature stability of asphalt binder by increasing the G^∗^ value by 25% and decreasing the phase angle (δ) by 3° on average compared to the control binder with the addition of 5% bio-oil; comparison among the three types of bio-oils showed that TB and PMB had the most significant effect on the asphalt, followed by the UTB.

The bio-oil used to modify a 1% SBS-modified asphalt binder resulted in a significant increase in the loss and complex shear moduli of bio-modified asphalt binder, which is desirable for anti-deformation ability and rutting prevention [200]. The rheological properties were also assessed; the bio-binder with 50% bio-oil showed higher complex modulus when the binder was RTFO aged, but the rest were almost identical to the conventional binder [125]. The polymer-modified bio-binder showed the highest stiffness, followed by the dewatered and original bio-binders. In addition, the original bio-binder had the least effect on the base binder [27,28]. 

A study investigated the properties of polymer-modified asphalt binder with the use of waste cooking oil in percentages up to 16% [93]. It was concluded that the effect of waste oil on the viscosity of the BMB is an adverse function; the increase in the oil content reduces the rotational viscosity. In addition, the viscosity–temperature relationship of the BMB was also determined; the increase in testing temperature reduces the rotational viscosity of the BMB. The mixing and compaction temperatures were found to be reduced through the addition of the bio-oil to the binder. It was observed that the average construction temperatures of asphalt mixtures decreased by approximately 1.8 °C with each 1% increase in bio-oil content in SBS-modified bitumen. Adding bio-oil enhances the low-temperature thermal cracking resistance of SBS-modified bitumen, negatively impacting its shear and rutting resistance.

In another study, an asphalt binder containing polymers such as SBS and polyethylene (PE) was studied by incorporating waste oil [90]. The study aimed to assess the performance of the bio-modified binder with different polymer content and 5% bio-oil. It was determined that adding waste oil to the SBS and PE-modified binder reduced the penetration by 13%. In contrast, the softening point was almost doubled, i.e., it increased from 46 to 90 °C, while ductility and viscosity were increased eight times the control binder. The aging effect on the different binders was also predicted. The SPI (softening point increment after aging) and VAI (viscosity aging index) were found to be increased when the modified binder was subjected to long-term aging. The penetration index and the ductility retention rate were found to be reduced after aging, which proves that asphalt binders containing waste oil and polymers have a good anti-aging ability.

A high complex modulus and low phase angle were obtained for the binder containing waste oil compared to the binder containing only polymers at higher temperatures and frequencies. The corresponding rutting parameters were higher. The binder with polymers had the lowest strain under creep loading. However, adding waste oil increased the strain, but the strain was way less than the base binder (no additives nor waste oil). Asphalt binders containing waste oil and polymers have lower temperature susceptibility and better storage stability [90]. The complex modulus was found to be enhanced by an average of 40% at different temperatures [89].

Another study used a lower dosage of 0.5% of bio-binder was added to two control binders, namely, PG64-22 binder and a polymer-modified form of the PG64-22 binder with 1.5% SBS to achieve a PG70-22 binder [102]. Asphalt binders were evaluated through BBR and MSCR tests. Table 10 shows the BBR results of the different tested blends at temperatures of −12 and −18 °C. The results showed that the critical temperature of the binder was reduced by adding 0.5% bio-additive by an average of 6%. 

In the MSCR test, the polymer-modified bio-binder showed slightly lower high-temperature stability than the other two binders. This was attributed to the low polymer fraction (only 0.4% by weight). The inconsistency between the two tests was attributed to the differences in loading magnitude and loading mode between the two tests. The improvement in the high-temperature performance was statistically significant [30].

#### 4.4.2. Rubber

Different types of rubber were used in the literature in combination with bio-oils, such as crumb rubber and tire rubber powder [204,205,206]. In a study that aimed at developing a bio-binder that can replace 100% of the petroleum-derived asphalt used in the construction of asphalt pavements, a bio-oil and crumb rubber (CR) with different percentages were utilized. In comparison to the base binder (AAM-1, PG64-16), the obtained critical temperatures (Tc) were similar to the bio asphalt binders after interacting with rubber [56]. The high-temperature binder grades are PG58 and PG64 for the bio-binders containing 10% and 15% crumb rubber. The study also investigated the use of bio-oil to modify the RAP binder. The high-, intermediate-, and low-temperature performances were evaluated using advanced rheological tests. The use of bio-oil in the base binder (viscosity grade VG30) softened the binder, which is evident from the increased penetration, decrease in softening point, and viscosity of the modified binder. The modified binder was more susceptible to aging, expressed by the increase in the aging index [87]. The G*/sinδ for the BR-15% at 10 Hz decreased by 71.7%, 73.1%, 74.8%, 75.7%, and 76.4% compared to the aged asphalt at the temperature of 52 °C, 58 °C, 64 °C, 70 °C, and 76 °C, respectively [105].

The addition of crumb rubber reduced the effect of bio-oil on the binder. The optimum binder content obtained through the Marshall test was found to be around 5.5% for all bio-binder contents. However, stability was found to increase with higher bio-oil content. The results obtained by Yang et al. (2018) showed that the increase in the oil content led to an increase in the Young’s and bulk moduli compared to the unaged asphalt binder. However, oil contents beyond 10% will eventually reduce less than the control unaged binder [31].

The bio-binder with lower rubber content exhibited better lower temperature performance than the base binder and the 15% rubber content modified binder. The blends with higher bio-binders showed lower temperature grades than the ones with higher rubber content. The findings of the study showed that non-crude petroleum binders could be developed effectively to replace typical paving grades of asphalt, such as a PG64-22 binder [56].

Asphalt binder was mixed with waste cooking oil, tire rubber powder (TR), and Bagasse ash (BA) in a study [89]. Physical tests were performed to determine the optimum contents, including penetration and softening points. The increase in WCO content in the asphalt binder reduces the softening point and increases penetration. Adding the tire rubber by 5% reduced penetration by 32%; the more TR content, the lower the penetration value. The 5% WCO and 15% TR, adding 8% BA as an additive, resulted in the optimum percentages of waste materials (W5TR15B8).

In a study, waste cooking oil was added to tire rubber powder. The bio-oil content was found to be at its optimum performance at 5% waste cooking oil content [91]. Using this percentage resulted in physical properties similar to the base binder’s (bitumen 80/100), and the rheological properties were within acceptable limits. The addition of tire rubber increased the viscosity of the asphalt binder, which had a higher oil content.

Waste cooking oil residue (WCRO) was used in a study to enhance the hot storage stability of rubber asphalt at different contents [77]. The complex modulus of the WCRO-modified rubber asphalt binder (HSSRA) was found to be lower than the rubberized asphalt binder at low, intermediate, and high frequencies in the unaged phase. This reduction was due to the softening induced by adding the WCRO. However, this reduction was not obvious after the short and long-term aging, so the aging resistance was enhanced. The aging resistance was improved as well. The results of the frequency sweep by DSR showed that DC bio-asphalt could improve high-temperature performance, whereas SH bio-asphalt could improve low-temperature performance [1].

The distinct physical and mechanical behaviors of the binder-rich phase (viscous, low density) and the rubber-rich phase (elastic, high density) caused dynamic asymmetry in the rubberized asphalt system, leading to phase separation. The diffusion of castor bio-oil into rubber (with the appropriate rubber–oil ratio) can balance these mechanical behaviors, resulting in dynamic symmetry in the rubberized asphalt system and improving its storage stability. Rubber pre-swelling also positively affected the elastic recovery of asphalt, thereby enhancing its resistance to permanent deformation or rutting. At a rubber–oil ratio of 1:1, the elastic recovery increased by 156% compared to asphalt containing non-swelled rubber. Rubber pre-swelling also improved the binder’s low-temperature cracking resistance, decreasing creep stiffness by 39% and increasing the m-value by 17%. Both the physisorption and chemisorption of bio-oils to rubber particles effectively enhance the properties of rubberized asphalt. Exploiting the synergy between these two mechanisms could further optimize rubberized asphalt performance. The study promotes the use of recycled rubber to enhance the sustainability of pavement construction [110].

Storage stability is crucial for modified asphalt binders, impacting performance stability during hot storage and transport [110]. Table 11 shows the segregation index (SI) of various rubberized asphalts used in this study. Among the five rubberized asphalts, SAR-M (surface activated rubber modified binder) had the lowest SI, followed by PsCR11-M (pre-swelled crumb rubber with CR to a bio-oil ratio of 1:1), with PsCR12-M (pre-swelled crumb rubber with CR to a bio-oil ratio of 1:2) having the highest SI. Comparing CR-M (CR-modified asphalt) and SAR-M rubberized asphalts, the SI reduction from 44% to 26% indicated that rubber surface activation positively affected rubber–asphalt interaction and compatibility, reducing the segregation of rubber and asphalt by 41%.

The low-temperature grade was reduced from −22 °C in the rubberized asphalt to −28 °C in the HSSRA, while the high-temperature resistance was decreased [77]. The addition of the oil, however, increased the m-value compared to the rubberized asphalt at lower temperatures, whereas the stiffness was increased. The fatigue resistance was enhanced due to the presence of WCO. However, the rutting resistance was due to the high rubber content [89].

Crumb tire rubber was mixed with WCO to create a waste rubber/oil (WRO) rejuvenator, which was then evaluated for its high- and low-temperature rheological properties before and after the aging of the rejuvenated binders [134]. It was found that WRO has the potential to be used as an asphalt rejuvenator. The WRO rejuvenator significantly improved the low-temperature properties of the aged binder while partially maintaining its high-temperature properties. With a 10% WRO rejuvenator, the performance grade of the artificially aged binder improved from 82-16 to 70-28. After aging, the continuous high- and low-temperature grading of the WRO rejuvenated binder remained relatively stable.

#### 4.4.3. Nano Particles

Nanomaterials are known for their use to improve the aging and adhesion properties of asphalt [207]. The incorporation of nanoparticles such as nano-silica, SiO_2_, CaCO_3_, etc., with bio-oil has been studied in multiple studies [129,207,208,209]. The rheological and pavement properties of asphalt binder modified with bio-oil and different types of nanoparticles, including SiO_2_, CaCO_3_, TiO_2_, Fe2O_3_, and ZnO, have been assessed. Adding the bio-oil to the asphalt binder increased the penetration of the bio-modified asphalt. However, adding the nanoparticles reduced the penetration due to the stiffening effect. Moreover, compared to the bio-asphalt binder, the decrease in penetration is greatest when nano-SiO_2_ is used and smallest when nano-CaCO_3_ is used. The dynamic stability ratio was found to be increasing with the increase of the bio-oil due to the decrease in the fracture strain. The low-temperature performance of nano-modified bio-asphalt is slightly diminished compared to bio-asphalt without nanoparticles. The trends observed with nano-SiO_2_ and nano-CaCO_3_ are more pronounced than those with other nanoparticles [129]. The MSCR test results also supported this finding by decreasing the non-recoverable creep compliance and increasing the percent recovery. It was determined that adding 5% of UTB decreased the J_nr_ value by 30% compared to the control binder, and so on with the remaining oil types. By comparing the DSR and MSCR test results, the untreated bio-asphalt binder showed slightly lower high-temperature stability due to high moisture content than the treated and the polymer-modified bio-asphalt binders. Rheological aging index analysis exhibited that all nano silica-modified bio-asphalts have a lower aging index as compared to the base asphalt [207].

Montmorillonite is another additive used to improve both the high-temperature rutting resistance and aging resistance of BMB blends [210,211,212]. Particles from submillimeter aggregates in the bitumen medium reduce their viscosity at low content and high temperatures and shift the solid–liquid transition point towards lower temperatures. In addition, these particles increase the bitumen’s stiffness, cohesion, adhesion upon cooling, but slightly worsen the low-frequency elasticity [211]. The button rock–montmorillonite modified bio-binder (RMBA) was optimized based on a series of tests to conclude that the mass ratio of water and bio-oil is 1:1, and the contents of bio-oil, rock asphalt, and montmorillonite are 7, 30, and 5%, respectively. Compared to the BB, the J_nr_ of RMBA decreased by 66.6% while the R% increased by 75.9% at a stress of 0.1 KPa. The stiffness of the RMBA was reduced by 0.87 times that of the BB. The antiaging performance of the RMBA was higher than the BB, as indicated by the G* aging index [210]. Adding montmorillonite into the BB is an effective practice to enhance the rutting resistance without compromising the fatigue performance of the binder [212].

#### 4.4.4. Warm Mix Additives

Warm mix additives (WMA) are widely used products that significantly reduce asphalt mixing and compaction temperatures [177]. The high-temperature performance of DSO–BMB modified with Sasobit as WMA was assessed in a study [179]. Sasobit has a stiffening effect on the high-temperature performance of the asphalt binders, which increases the rutting resistance significantly [213]. Therefore, the addition of DSO reduced the G*/sinδ. Researchers suggested that DSO content over 1.5% is not feasible due to the reduction in the rutting resistance [179]. The low-temperature performance of asphalt was enhanced by the addition of a Sasobit and soybean oil composition when added to a styrene-butadiene rubber (SBR) polymer asphalt binder, as the stiffness was reduced at testing temperatures [214]. In addition, short- and long-term aging resistances were significantly enhanced by the addition of Sasobit and soybean oil.

The rutting performance of different bio-modifiers was evaluated in a study [101]. The recovery percent (R%) at 3.2 kPa stress level did not show a clear trend for the different additives and binders. For example, the reactor product (RP) additive induced an increase of 35% in the R% for the PG58-28 binder; however, there was a reduction of 8% in the PG64-28 asphalt binder. On the other hand, RP-modified PG58-28 maintained a high-grade traffic level compared to the conventional PG58-28. The rest were reduced to standard-grade traffic levels, and the PG64-28 asphalt binder, which has a very high-grade traffic level, was also reduced. The addition of the RP did not change the classification, but the rest of the binders in this group were reduced to high-grade traffic levels, except the CI and FA. Further details on a lower dosage of IDB, FP 1, and FB 2 were compared in a later study [102]. An average reduction of 29% and 8% for the modified PG64-22 and 74-22, respectively, was obtained for all modifiers. The MSCR classification of the binders concluded that the 0.5% IDB-PG64-22 modified binder could maintain the high-grade traffic level. However, FP1 and 2 were rated as standard-grade traffic levels. On the other hand, the IDB addition to PG70-22 reduced the traffic grade to standard, and the rest maintained the high-grade traffic level.

Asphalt binder mixed with graphene oxide (GO), i.e., a nano-sized material sourced from graphite, which is rich in oxygen functional groups, Sasobit, and WCO with contents of 0.05, 3, and 5%, respectively [37]. The use of Sasobit and WCO was compared to each other and had a synergistic effect on the modified GO asphalt binder (HMAB-2). The effect of the Sasobit as a warm mix additive compared to the WCO as a rejuvenator resulted in similar mechanical properties such as mixing and compaction temperatures. Using both modifiers together increased G* at higher testing frequencies; however, the modified binder was slightly higher at lower frequencies. The associated traffic level for the Sasobit-GO modified asphalt was extreme; however, the addition of the WCO reduced the traffic level to be heavy as per the requirements of the AASHTO M332. The combined use of 0.05 wt.% GO, 3 wt.% Sasobit, and 5 wt.% WCO significantly enhanced asphalt binders by reducing viscosity, improving elastic recovery, increasing high-temperature stiffness, enhancing low-temperature relaxation, and boosting cracking and fatigue resistance. These modifications resulted in asphalt binders with balanced performance at high and low service temperatures, making them suitable for use in various regions to achieve optimal road performance.

#### 4.4.5. Other Materials

Polyphosphoric acid (PPA) was added to the bio-modified binder at a dosage of 1.5% in one study. The addition of PPA resulted in a significant increase in complex modulus, which, in turn, increased the elasticity of the bio-modified binder. This practice indicated promise for mitigating the negative effect of adding the bio-binder on the rutting resistance [52]. PPA was chosen to partially replace SBS in the bio-binder, with a maximum replacement level of 1.5% of the total binder content, as any additional replacement does not enhance performance [215]. The bio-binder content had a small effect on the elastic recovery; however, increasing the PPA content reduces the modified binder’s recovery. Higher segregation was obtained at more than 10% BB content when the PPA content was 0.5%. 

A bio-oil/lignin composite modified asphalt (OLMA) was used to study the rheological and aging properties in a study [216,217]. Different composites were prepared (e.g., BO5L10 represents 5% bio-oil and 10% lignin). The G* modulus master curve of all tested blends. The DSR tests showed that OLMA was improved at the high-temperature, fatigue, cracking, and relaxation performance of asphalt. The OLMA can reduce the production of oxidation functional groups during aging. The optimum dosage of 10% bio-oil and 20% lignin was determined [216].

### 4.5. Performance of Asphalt Mixtures

#### 4.5.1. Laboratory Investigation of Asphalt Mixtures

Incorporating BB, asphalt binder, and many other additives, such as crumb rubber, RAP, etc., is highly studied. This section discusses the different results of HMA laboratory testing throughout the literature. The Hamburg rut test (AASHTO T 324) showed a slight increase in the rut depth for the 2% BMB compared to the nonmodified binder. However, the researchers suggested further in-depth studies are needed to evaluate higher bio-binder contents in asphalt mixtures [25]. The Marshall test results showed that the modified binder’s optimal asphalt aggregate ratio was slightly reduced compared to the base binder (4.47 to 4.45). The modified mixture exhibited the lowest Marshall stability (MS) and flow value (FL) due to its high void ratios, resulting in reduced strength and increased deformation [9].

The radius of gyration of modified asphalt showed an abrupt change at 333 K, indicating a potential internal structural change and the occurrence of a chemical reaction [98]. Adding oil reduces the fracture strength of the asphalt binder, thereby diminishing its low-temperature cracking resistance. In addition, Thermal Stress Restrained Specimen Test (TSRST) test results for the asphalt mixture verified the findings that the fracture temperature of the bio-modified asphalt mixture was much lower than the original asphalt mixture [155]. Reduction in the fracture energy at intermediate temperatures compared to the base binder was observed, which indicates a reduction of fatigue resistance [157].

The addition of the BMB increased the failure strokes by 27% for the 5% BMB and 179% for the 10% BMB. However, the difference became more evident after 120 h of conditioning, where it increased by 260% and 446% for both BMBs compared to the control binder [180]. This means that the BMB was able to sustain higher strains before failure. The addition of 5% BMB showed very little change in the failure load, while the 10% BMB showed an increase of 17% compared to the control binder before the conditioning. After the conditioning, both BMBs showed around 38% change in the failure load compared to the control binder. According to Oldham and Fini (2020), the calculated contact angle for the BMBs was calculated after the binder samples were conditioned in 80 °C 5 mL deionized water for 2 h, then placed in a room temperature deionized water bath for 5 min. The samples were then dried using nitrogen gas. The contact angle was measured using a goniometer before and after water exposure. The contact-angle moisture susceptibility index (CAMSI) was then calculated. The dry binders (control and BMBs) did not show significant differences in the captured images. However, after water exposure, a significant change was obtained. The contact angle for the control binder jumped to 133°, 85° for 5% BMB, and 41° for the 10% BMB. In other terms, the lower values of the CAMSI for the BMBs indicate the improvement in the moisture resistance of the binder compared to the control binder by 70% and 90% for the 5% and 10% BMBs, respectively. The tensile strength ratio of the 5% BMB mixture was tested as per AASHTO 283. The samples were first saturated to 70–80%, then subjected to freezing at −18 °C; the samples were then thawed at 60 °C for 24 h. The testing was conducted at 25 °C. Including the RAP in the mixture enhanced the tensile strength ratio (TSR) for the binders despite their modification type. The 5% BMB had higher TSR values than the control with 0% RAP; however, at 15% RAP content, the TSR value was a bit higher for the control mix than the BMB. Again, at 45% RAP content, the TSR for the BMB mix was higher than that of the control mixture. The results of TSR for different blends are shown in Table 12. The rutting performance per the Hamburg wheel tracker test showed weaker performance for the BMB mixture than the control mixture. Regarding moisture susceptibility, the BMB mix showed improved stripping inflection point values despite having a higher rut depth in certain cases. 

The rut depth results of asphalt mixtures containing high RAP and varying oil contents showed that the maximum rut depth was 5.68 mm, observed in the mixture with 5% WCO, 25% PG64-28, and 70% RAP at 64 °C. However, only two modified binders (5% SOY_25% PG64-28_70% RAP and 5% WCO_25% PG64-28_70% RAP) and their respective HMA mixes produced fatigue cracking resistance similar to that of the virgin binder and virgin mixes. Overall, modifying the RAP binder was found to be necessary for using high percentages in HMA. However, using bio-oils alone did not produce HMA mixes with fatigue and low-temperature cracking resistance comparable to control mixes [218,219].

Another study evaluated the low-temperature performance of bio asphalt mixture containing up to 45% RAP and 10% bio-oil [220]. The effect of the bio-oil on the asphalt binder was clear at a temperature of 105 °C; this effect was reduced and became less significant at higher temperatures. The stiffness values were decreased by 1.1, 8.9, and 11.4% in the case of swine manure, corn stover, and wood pellets bio-modified binders, respectively, but it was increased by 2.3% in the case of miscanthus pellets modified binder. The m-value was affected more significantly for the swine manure, corn stover, miscanthus pellets, and wood pellets binders, having 6.9, 11.3, 8.9, and 10.4% increased m-value compared to that of the control binder. In addition, bio-asphalts improved mixture fracture energy regardless of RAP content compared with HMA due to reduced production temperatures. 

The low-temperature properties of the 5% BMB blended with different RAP contents were investigated by Hill et al. (2013) in asphalt binder and HMA through performing multiple tests, including the Disk-Shaped Compact Tension [DC(T)], Superpave Indirect Tension (IDT), and Acoustic Emission (AE) [5]. The DC(T) test at −12 °C indicated that the crack-mouth opening displacement (CMOD) fracture energy (J/m^2^) for the bio-binder was 4.2% higher than the virgin binder with 0% RAP content. However, adding 15% RAP to the HMA reduced the fracture energy by 8.0% compared to the virgin HMA. The value was then increased by 11.5% compared to the virgin binder with 15% RAP.

Adding the bio-binder to the HMA mixture with 45% RAP content resulted in an 18.7% increase in fracture energy compared to the virgin binder with 45% RAP. The BMB mixtures produced better low-temperature creep compliance even in the case of 15% and 45% RAP contents. This was explained by the high m-value for the BMB mixtures compared to the conventional HMA. As a result, the stress relaxation characteristics were found to be enhanced when the BMB was introduced in conjunction with RAP. Through the AE test, the embrittlement temperature (T_EMB_) was found to be reduced by 23.7% when the BMB mixture was tested compared to the virgin HMA. Adding 15% RAP to the virgin HMA reduced the T_EMB_ by 5.7%; however, a further 24% was reduced when the BMB was introduced to the mixture. The same effect was obtained when the RAP content was higher. The maximum energy event (Tmax) temperature was also obtained. The resulting T_max_ was 51.1% higher in the BMB mixture than the virgin HMA. However, despite the increase in the RAP content, the T_max_ was almost similar. Therefore, the transition range size (T_EMB–_T_max_) was higher in the BMB mixtures compared to the conventional HMA. 

A further study also assessed the same bio-oil types and contents but for asphalt mixtures [29]. The rutting resistance, fatigue performance, dynamic stiffness, and tensile strength of asphalt mixtures were evaluated using the asphalt pavement analyzer (APA) test, four-point beam fatigue test, dynamic modulus (|E*|) test, and indirect tensile (IDT) strength test, respectively. The bio-modified mixtures exhibited a higher dynamic modulus (|E*|) compared to the control mixtures, with average increases of 8.1%, 6.1%, and 10.3% for the original bio-oil (OB), dewatered bio-oil (DWB), and polymer-modified bio-oil (PMB) modified asphalt mixtures, respectively. This increase was attributed to the bio-oil’s higher stiffness than the asphalt binder. Additionally, the |E*| increment was more significant at low reduced frequencies (high temperature and low frequency) than at high reduced frequencies (low temperature and high frequency), indicating that bio-oil significantly increases the stiffness of asphalt mixtures at relatively higher temperatures. Consequently, the PMB-modified asphalt mixture was expected to exhibit better rutting resistance than the other two types of asphalt mixtures. However, the IDT strength of the modified mixtures was lower than that of the control mixture by 16.7%, 18.3%, and 9.3% for OB, DWB, and PMB, respectively. The test results showed that adding bio-oils significantly improves fatigue performance, has no significant effect on rutting performance and dynamic modulus, but slightly reduces tensile strength. Additionally, the inclusion of polymers in the bio-oil enhanced asphalt mixture performance, with PMB-modified asphalt mixtures outperforming the other two mixtures.

On the other hand, bio-oil blended with different tire rubber sources and cryogenic and ambient rubbers were used to modify PG58-28 and PG64-22 asphalt binders at 20% BMB content in asphalt mixtures [57]. The study concluded that the modified mixtures performed very well in all carried out tests, and the mixtures are stand-out rutting, early fatigue cracking distresses, moisture damage, and low-temperature cracking. The combination of the binders and bio-oil affected the overall performance grade of bio-binders. Additionally, the study showed that the mixture dynamic modulus results do not necessarily accord with the asphalt binder test results [221].

The applicability of the DSO in asphalt mixtures increased the rutting susceptibility of mixtures. The addition of the RAP limited such reduction. This is explained by the rut depth results that were found to be increased at lower contents of RAP and higher contents of DSO. The ITS test was conducted on saturated and unsaturated samples to determine DSO’s moisture susceptibility and effect. The results showed that the addition of the DSO increased the TSR results for blends with 20% RAP, and DSO content slightly increased when DSO% was 5%; however, it was reduced again when the content was 10%. Blends with 30% and 40% RAP content were intended to increase the TSR results at both oil content continuously. The fatigue life was improved by 15% when 10% DSO was added to the 20% RAP binder. Test results showed that the asphalt mixture containing 90% RAP and a bio-oil rejuvenator had a 37% increase in fatigue life and a 4% improvement in moisture resistance [192]. Results suggest the bio-binder has similar moisture damage resistance to the neat asphalt binder and higher resistance than the highly modified asphalt binder, providing a first indication for the potential use of this bio-binder as a 100% asphalt binder replacement [222].

Bio-origin additives used for bitumen fluxing hold significant potential for RAP technology, enhancing the blending of RAP with virgin materials and binders during RAP rejuvenation. Future studies should conduct an in-depth investigation into the application of bio-origin additives in asphalt mixtures [106]. The performance of asphalt mixtures containing 30% RAP at 2.5% bioagent content was determined further [63]. A reduction in the |E*| values was obtained with mixtures containing bioagent compared to the control mixture (asphalt binder with 30% RAP) and vice versa for the phase angle because the bioagent demonstrates lower stiffness of the binder. From the Thermal Stress Restrained Specimen Test (TSRST), the failure temperature was found to be reduced from −20 °C for the control asphalt mixture containing 30% RAP to −25 °C for the bio-modified asphalt mixture. However, the cryogenic stresses, i.e., the stress before sample breaking, were increased by 6% compared to the control asphalt mixture.

A mixture of 5% waste cooking oil and modified asphalt bitumen was prepared and tested in a study [165]. The waste cooking oil was added to the binder in both its treated and untreated forms. Creep stiffness and voids characteristic after aging of the mixture were determined. The flow value was higher for the controlled samples, while for the samples with untreated and treated waste cooking oil, the values were less than the controlled ones. The creep stiffness values were increased when treated waste cooking oil was used in the asphalt mixture. However, the result was exactly the opposite when untreated waste cooking oil was used. In a review, the effect of waste cooking oil and waste engine oil in asphalt binder and mixtures has been researched [136]. Since the oil could reduce the viscosity of the asphalt binder, the reduced rutting performance raised a main issue in the modified asphalt. These effects depend on the amounts used in asphalt mixtures and materials. A comparison between the cold and hot asphalt mixes indicated that the performance in terms of stability, strength, and the bonding between aggregate showed that the hot mix asphalt, where the oil was integrated with RAP, offered stiffness reduction and improved resistance to cracking. The amounts of RAP binder and waste oil, in addition to the temperature, are the main parameters that significantly influence the performance. 

The low-temperature improvements can occur with the addition of bio-derived material additives to the asphalt binder [100]. Different additives were added to WMA to investigate their low-temperature performance characterization, which is more challenging due to the response of the aggerates in mixtures. Therefore, the semi-circular bend (SCB) test was conducted to characterize the fracture properties of modified mixtures. The average fracture energy of the modified binders at a temperature of −18 °C was found to decrease compared to the control binder (PG58-28) by 7% for the IDB, 19% for the FP1, 21% for the CI, 30% for the FP2, and 31% for the RP. However, an increase of 32% was due to adding FA. Similar trends were obtained for the fracture toughness and stiffness values. A later study investigated the same test but at different temperatures. It was evident that the fracture energy increases at high testing temperatures [102]. The stiffness results are that as temperature increases, the stiffness decreases. However, the PG70-22 binder does not exhibit this trend between −24 and −12 °C when modified with 0.5% FP 2. PG70-22 binder modified with 0.5% FP 2 does not differ statistically from PG70-22 binder at −24 or −12 °C, probably due to the material reaching the glass transition zone.

Moisture damage resistance testing results indicated that DC bio-asphalt significantly increases moisture susceptibility [1]. The immersion Marshall and freeze–thaw split test results for base asphalt, 10% DC bio-asphalt, and 15% SH bio-asphalt showed a notable increase in moisture susceptibility. This resulted in a sharp reduction in Marshall stability after 48 h of water immersion and a loss of tensile strength following freeze–thaw cycles. The Marshall stability of the asphalt mixture with 10% DC bio-asphalt before conditioning decreased by 29% compared to the base asphalt.

The dynamic stability (DS) and rutting depth of mixtures made with BA, SBS, BBA, PMB, and chemically modified bio-based asphalt (CMB) showed that most mixtures containing BB have higher DS values than the BA mixture, except for the 15% CMB mixture. Comparing the BA and BBA mixtures, adding BB improved rutting resistance, which was consistent with the results from physical properties, DSR, and RV tests. The SBS-modified asphalt mixture demonstrated higher DS and lower rutting depth. Flexural–tensile strain (ϵB) measurements indicated that the ϵB of mixtures with 10% BBA and 15% BBA were 5.1% and 9.1%, respectively, lower than that of the BA mixture. Since higher ϵB is beneficial for low-temperature performance, this suggests that adding BB to BA weakens low-temperature cracking resistance, aligning with the BBR test results [84].

Resins (Damar) were used to modify the asphalt binder in the pavement mixture. In a study, laboratory tests were performed on the modified asphalt concrete mixture, including unconfined compressive strength, indirect compressive strength, and permeability [40]. The maximum stability of mixtures was determined to obtain the optimum bitumen content. It was determined that the asphalt mixture with higher resin content showed a stability improvement compared to the rest of the binder. The ITS (indirect tensile strength) and UCS (unconfined compressive strength) of the modified asphalt mixes were increased by 65% and 31% with the addition of 2.5% of resin, respectively. The failure loads were found to be increased linearly. 

A bio-binder produced from agriculture and forestry residues was mixed with ground tire rubber in a study to be compared with conventional asphalt binder sourced from crude petroleum [223,224]. The blend of the PG64-22 asphalt binder with 20% cryoMBO was selected to produce asphalt mixes with a nominal aggregate size of 9.5 mm, as this binder exhibited the best overall performance across various types of distress. The global performance of the mixtures was evaluated through moisture susceptibility, flexural fatigue cracking, dynamic modulus, rutting resistance, and low-temperature fracture tests. The mixtures with the chosen asphalt blend performed very well in all tests. Consequently, these mixtures are not expected to suffer from rutting or early fatigue cracking and should not be susceptible to moisture or low-temperature cracking. Laboratory observations indicated that bio-binders require agitation to prevent phase separation, necessitating special tanks with agitation systems. Based on laboratory results, GTR particle sizes larger than #80 mesh in bio-binders are not recommended to avoid stability and phase separation issues. In addition to using special tanks for transportation, sufficient commercial production of fractionated bio-oil is necessary for the technology to be readily applicable. Researchers believe ample agricultural and forestry residues exist for commercial fractionated bio-oil production. To persuade transportation agencies to approve using higher quantities of bio-oil as an alternative binder for pavements, the excellent laboratory performance of these new mixtures should be validated through pavement trials after asphalt plant production. Using higher quantities of bio-oil in pavements would offer technological, environmental, and economic benefits. This report summarizes the successful development of a bio-binder derived from renewable materials, representing green technology.

The addition of refined engine oil bottoms (REOB) improved the lower continuous PG temperature of the asphalt binders evaluated [225]. Regarding rutting performance, no stripping cracks were observed in all mixtures. The fracture temperature increased with the addition of REOB; 15% of REOB has a lower fracture temperature than lower oil contents. The fracture strength and thermal stress decreased with oil addition [225]. In further review, the bio-oil can significantly improve the quantity and quality of asphalt mixture with modification of Polymer Modified Bitumen (PMB) [26,43].

#### 4.5.2. Field Results

The site live trials concluded that the Vegetex 50 and 40/60 binder performed similarly regarding texture depth and surface regularity. The Vegetex 50 mixture aged 20% less than the 40/60 mixture, which means that the long-term performance and durability of the asphalt composites can be enhanced [148,149]. Through the calculated Flexibility Index (FI), it was found that increasing the FI increases the fatigue resistance of the binder [96]. 

A study evaluated six road sections to determine the causes of surface dressing failures [226]. Asphalt pavements contained modified asphalt with fish and rapeseed oil. Full rheology for the asphalt binder was conducted. Critical issues, including bleeding, bonding, or low shear resistance, were observed in the different samples. The visual inspection of the samples from different sections revealed that the sections that contained fish oil with a bitumen stabilized layer were identified without bleeding compared to similar sections with a stabilized layer where bleeding occurs; the most important factor is that these sections have traffic levels significantly lower than the other sections. The bonding failure between aggregates and binders was attributed to the solubility issues of the bio-oil, which coated the aggregate particles and prevented proper bonding with the binder, resulting in the binder draining down. Rheology tests showed that the binders became much stiffer after recovery (including mixing, laying, field aging, and laboratory recovery), likely approaching the stiffness of the unaged reference bitumen (Binder N). Although traditional testing makes it difficult to find a comparable basis due to the lack of a common test method in the literature, a comparison based on viscosity tests under specific conditions was conducted, as shown in Table 13. Table 14 shows a summary of the performance parameters reported in different studies for BMBs at a 5% oil content level.

## 5. Conclusions

This research was conducted to provide a comprehensive review of the applicability of bio-oil originating from different sources in modifying asphalt binders and asphalt mixtures. A wide range of research was conducted to evaluate the performance of the bio-oil-modified binder (BMB). The main conclusions of this research are:

1. Effect on Rheological Properties: Bio-oil modifies the rheological properties of the asphalt binder, improving its performance against pavement distresses.
2. Viscosity Increase: The bio-binder increases the viscosity of the asphalt binder at high service temperatures.
3. High-Temperature Performance: Adding bio-oil reduces the high-temperature complex modulus and phase angle, lowering the high-temperature PG of the asphalt binder. This benefits stiff binders, such as those modified with polymers/rubber or RAP. However, higher bio-oil content can increase rutting susceptibility.
4. Feasibility as a Modifier: Bio-oils are feasible alternatives or modifiers for asphalt binders due to the enhancements introduced to the binder or mixture.
5. Penetration and Rejuvenation: Bio-oil increases binder penetration and reduces stiffness, making it an excellent rejuvenating agent for aged binders (RAP).
6. Reduced Softening Point and Viscosity: Bio-oil addition reduces the softening point and rotational viscosity, leading to lower mixing and compaction temperatures.
7. Aging Susceptibility: The aging mechanism of the modified binder changes with bio-oil addition, increasing susceptibility to aging.
8. Low-Temperature Performance: BBR tests indicate that bio-oil extends the range of intermediate and low-temperature PG by reducing the minimum operational temperature.
9. Fatigue Performance: Proper bio-oil content can extend the low-temperature range without compromising binder consistency, as shown by DSR and BBR fatigue parameters.
10. Fatigue Cracking Resistance: LAS tests demonstrate that bio-oil enhances fatigue cracking resistance, indicated by increased Nf values.
11. Rejuvenation Efficiency: Bio-oil effectively reduces the stiffness of aged/RAP binders, making it suitable for recycling purposes.
12. Optimal Oil Content: The optimal bio-oil content depends on the binder’s function and oil properties. Low dosages enhance low-temperature performance, while high contents rejuvenate aged/PAV binders.
13. RAP Technology: Bio-origin additives improve RAP technology by enhancing the blending of RAP with virgin materials and binder rejuvenation.
14. Rejuvenating Agent: Bio-oil is a suitable rejuvenating agent for aged asphalt binders in bituminous pavements.
15. Optimal Dosage: A 5% bio-oil content is optimal for rejuvenating aged binders, offering better rutting and fatigue performance than virgin binders. Bio-oil is capable of changing the rheological properties of the asphalt binder, which will improve the performance against pavement distress.

The studied literature showed a deficiency in pointing out a comprehensive assessment of the performance of asphalt binder and asphalt mixtures modified with bio-oil. The authors recommend studying further sources using waste materials such as animal manure or organic waste.

## Figures and Tables

**Table 1:** Comparison of thermochemical processes for bio-oil production [19].

Method	Treatment Condition/Requirement	Reaction Mechanism/Process Description	Technique Feasibility	
			Pros.	Cons.
Flash/fast pyrolysis	Relatively high temperature (450–500 °C); a short residence time (<1 s); atmosphere pressure; drying necessary	The light small molecules are converted to oily products through homogeneous reactions in the gas phase	High oil yield; up to 80% on dry feed; lower capital cost	Poor fuel quality obtained
Hydrothermal liquefaction (HTL)/liquefaction/hydrothermal pyrolysis	Lower temperature (300–400 °C); longer residence time (0.2–1.0 h); high pressure (5–20 Mpa); drying unnecessary	Occurs in an aqueous medium, which involves complex sequences of reactions	Commercialized already better quality of bio-oil obtained (high heating value, low moisture content)	Relatively low oil yield (20–60%); need high pressure equip, thus higher capital cost

**Table 2:** Recycled blends produced by Ingrassia et al. (2020) [73].

Blend *	Composition (by Weight)	Description
A + RAP	71% A + 29% RAP	Recycled binder formed by a severely aged bitumen blended with a fresh bio-binder
B + RAP	71% b + 29% RAP	Recycled binder in which the aged and the fresh binders are composed of 100% bitumen
A + bio-RAP	62% A + 38% bio-RAP	Recycled binder in which the aged and the fresh binders are bio-based (both contain 10% by weight of wood bio-oil)
B + bio-RAP	62% B + 38% RAP	Recycled binder formed by a severely aged bio-binder blended with a fresh bitumen.

* A: Composed of 10% by weight of a wood-based bio-oil, B: Bitumen with physical and mechanical properties similar to A.

**Table 4:** Details of common standard laboratory tests conducted to evaluate the performance of asphalt binder blends.

Material	Performance Parameter	Test	Aging Level	Standard	Temperature Range (°C)
Asphalt Binder	Conventional Properties	Penetration Softening point	Unaged	ASTM D5 [112] ASTM D36 [113]	25 N/A *
	Workability	Rotational viscosity (RV) Temperature susceptibility	Unaged and RTFO	ASTM D4402 [114] ASTM D2493 [115]	135
	Rutting Resistance	G*/sinδ Multiple Stress Creep Recovery (MSCR)	Unaged and RTFO	ASTM D7175 [116] AASHTO T350 [117]	42–84 High PG
	Fatigue Resistance	G*sin δ Linear Amplitude Sweep (LAS)	PAV	ASTM D7175 [116] AASHTO T391-20 [118]	31–13 Intermediate temp
	Low Temperature Cracking	BBR	PAV	ASTM D66 48 [119]	0–(−24)
	Overall Performance	Frequency sweep		AASHTO T315 [120]	10–70
	Storage Stability	G*/sinδ Softening point	Unaged	ASTM D7175 [116] ASTM D36 [113]	163 -
Asphalt Mixture	Stiffness	Dynamic modulus	-	ASTM D3497 [121]	(−10)–54
	Rutting	Flow number Hamburg wheel test	-	AASHTO TP 79 AASHTO T 324 [122]	54 60
	Strength	Indirect tensile strength (IDT)	-	ASTM D6931 [123]	
	Fatigue	Four-point beam fatigue test Three-point bending cylinder	-	ASTM D8237 [124]	

* N/A: Not Applicable.

**Table 5:** Specific gravity of different bio-oil types from different studies.

Bio-Oil Type	Waste Vegetable Oil ^1^	Waste Vegetable Grease ^1^	Organic Oil ^1^	Distilled Tall Oil ^1^	Aromatic Extract ^1^	Waste Engine Oil (WEO) ^1^	Palm Kernel Oil Polyol ^2^	Black Alder Wood ^3^	Castor Oil ^4^	Swine Manure ^5^
Specific Gravity	0.917	0.924	0.947	0.950	0.995	0.872	1.114	1.2	0.88	1

Data obtained from sources ^1^ [108], ^2^ [99], ^3^ [30], ^4^ [110], ^5^ [25,137].

**Table 6:** Consistency and viscosity results of unaged BMB blends. Data obtained from source [143].

BMB Blend	Penetration	Softening Point (°C)	RV@135 °C (Pa.s)	Mixing Temperature (°C)	Compaction Temperature (°C)
0.0% DSO-BMB	54	49.93	0.583	159–165	144–147
1.5% DSO-BMB	66	49.75	0.381	152–158	136–139
2.5% DSO-BMB	84	45.03	0.323	148–154	132–136
3.5% DSO-BMB	102	43.5	0.323	147–153	131–135
4.5% DSO-BMB	117	42.38	0.298	146–153	130–134
5.5% DSO-BMB	139	41.03	0.263	142–148	127–131

**Table 7:** Differences between top and bottom in |G*|/sinδ results for different bio-binder content. Data obtained from source [125].

Bio-Binder Content	Location	|G*|/sinδ
2%	Top	1.56
	Bottom	1.68
8%	Top	1.75
	Bottom	1.66
25%	Top	2.30
	Bottom	2.42
50%	Top	2.27
	Bottom	17.7

**Table 8:** BBR creep stiffness results of 5% modified binder at different testing temperatures. Data obtained from source [139].

Parameter		m-Value			BBR Creep Stiffness (MPa)		
Temperature (°C)		−12	−18	−24	−12	−18	−24
Asphalt Binder Type	PG64-22	0.364	-	-	240	-	-
	5% Bio-binder	0.383	0.392	0.412	260	290	310

**Table 9:** Aging index of BMB containing 25% TLA. Data obtained from source [162].

Oil Content (%)	Penetration		Softening Point		Viscosity	
	RTFO/Unaged	PAV/Unaged	RTFO/Unaged	PAV/Unaged	RTFO/Unaged	PAV/Unaged
0	0.78	0.46	1.07	1.20	1.77	3.25
3	0.77	0.45	1.08	1.22	1.83	3.39
6	0.75	0.44	1.08	1.23	1.87	3.79
9	0.73	0.43	1.09	1.24	1.94	4.33
12	0.69	0.39	1.10	1.27	2.02	4.98

**Table 10:** Average BBR results for different blends. Data obtained from source [102].

Temperature (°C)	−12		−18		Critical Temperature (°C)
Blend ^1^	Stiffness	m-Value	Stiffness	m-Value	
Control (PG64-22)	144.4	0.332	246.7	0.272	−25.2
IDB	125.5	0.346	226.7	0.29	−26.9
FP1	154.7	0.337	312.7	0.288	−26.5
FP2	149	0.34	271.7	0.288	−26.58
Control (PG70-22)	117	0.341	236	0.267	−25.31
IDB	129.3	0.344	228	0.28	−26.12
FP1	118.3	0.369	232.7	0.314	−29.52
FP2	124.3	0.346	257.3	0.297	−27.63

^1^ IDB is isosorbide distillation bottoms, FP1 and FP2 are bio-based WMA additives.

**Table 11:** Segregation index of the rubberized asphalt binder. Data obtained from source [110].

	CR-M	SAR-M	PsCR21-M	PsCR11-M	PsCR12-M
Segregation Index (%)	44	26	55	35	57

**Table 12:** Tensile strength ratio test results for control binder, BMB, AD1, and AD2 with 0%, 15%, and 45% RAP. Data obtained from source [180].

RAP Content (%)	Tensile Strength Ratio (%)			
	Control	BMB	AD1	AD2
0%	70	75	68	80
15%	80	78	75	90
45%	72	80	65	85

**Table 13:** Superpave binder grading [226].

Binder	N			K8			K9		
Viscosity at135 °C Max, 3 Pa.s	0.2			-			-		
PG, max pavement design temperature, °C	46	52	58	46	52	58	46	52	58
Dynamic shear (10 rad/s) G*/sinδ, Min 1.00 kPa	4.48	2.00	0.93	0.44	0.22	-	0.74	0.36	-
After RTFOT									
Mass loss, Max 1%	0.6			2.85			1.71		
Dynamic shear (10 rad/s) G*/sinδ, Min 2.20 kPa	10.88	4.5	1.98	2.29	1.10	-	2.38	1.08	-
After PAV (100 °C)									
Temperatures	13	10	7	7	4	1	4	1	−2
Dynamic shear (10 rad/s) G*sinδ, Max 5000 kPa	4170	6320	-	3490	3660	5530	2860	4470	6540
Min pavement design temperature, °C	−12	−18	−24	−24	−30	−36	−24	−30	−36
After PAV									
Creep stiffness (60 s)									
S, Max 300 MPa	61	178	Sample Broke	-	264	563	m-value < 0.1		
m-value Min 0.300	0.434	0.358		-	0.363				
Estimated performance grade	PG52-28			Failed			Failed		

**Table 14:** Summary of performance parameters for different BMB.

[Reference]	Bio-Oil Properties		Asphalt Binder Properties		Bio-Modified Asphalt Binder Properties †						Bio-Modified Asphalt Binder Performance ‡	
Measurement Parameter	Bio-Oil Type	Extraction Method	Base Binder (PG or Pen)	Additives	Consistency			Rheological ** Properties			Rutting Resistance	Fatigue Resistance
					Penetration	Softening Point	Rotational Viscosity *	High Temperature	Intermediate Temperature	Low Temperature	|G*|/sinδ or Recovery	|G*|sinδ or LAS
[1,84]	Corn oilCastor oil		PEN 90		7.1 and 31	1.1 and −5.4						
[4,180]		Thermochemical conversion	PG64-22	RAS			−30	−50			Jnr: −50 R%: −9	
[5,220]	Swine manure (SM) Woods (WP) Corn stover (CS)		PG64-22	RAP			SM and CS: −6.25 WP: −4.2			SM: 0 WP: −12 CS: −10		
[25,50,52]	Swine manure	Thermochemical liquefaction	PG64-22				−35	−30		−5		
[63]	Rapeseed methyl esters	oxidation reaction promoter	Virgin Bitumen 35/50					−88.2				
[64,143]	Date seed oil	Soxhlet	PEN 60/70	-	160	−18	−55	−65	−74	−50	−67	−62
[66]	Pongamia oil		Viscosity grade-20				−70				−75	
[69,191,192]	Date seed oil	Soxhlet	PG64-22	RAP			−60 no RAP			−16	|-66 Jnr: +21 R%: −15	Nf 2.5%
[72,73,103]	Wood		50/70 penetration grade	RAP	38.4	−5.4	Mixing and compaction temperature: −6.3				−34.4	−28.1
[76,186]	Waste cooking oil		AGED BITUMEN 60/70		200	−22.4						
[81]	Soybean oil Niger seed oil		PAV PEN50				-33	-64				−45
[83]	Waste edible vegetable oil		PAV PEN 60–80 and 40–60 and 40–60 (SBS)		A: 187.5	A: −13 B: −11.7 C: −13.2	A: −35.7 B: −44.7 C: −46.5	A: −83.3 B: −72.1 C: −55.6			A: −75 B: −55 C: −45	
[86,200]	Wood chips	Industrial oil	PEN 50	SBS	5	−2		−20.8				
[87]	Waste wood	fast pyrolysis	Viscosity grade 30 RAP binder		200	−16.9	−30				Jnr: +45.5 R%: −68.4	Nf: +305%
[89]	Waste cooking oil		Virgin Bitumen 60-70	Tire Rubber powder (15%) Bagasse ash (8%)	42.6	4.3	188.9	37.7				−14.3 stiffness: −45.9
[90]	Waste oil		PEN 80/100	SBS polyethylene (PE)	70.6	−3.2	−31.9	−55.3				
[106]	Rapeseed		PG70-16 PG64-16 PG58-22		−26.5			−87 −66.7 −85				
[128,227]	Corn stover	fast pyrolysis	PEN 80/100	crumb rubber powder			24.3				R%: 23.4	
[139]		Thermochemical liquefaction	PG64-22				−32.5			−10		
[146,155]	Waste oil		PG64-22							−60	Jnr: 20.7	Nf: 71.4
[158]		Pyrolysis process	PG64-22	RAS RAP Sasobit						10		
[182]	Swine manure		PG52-28				−9			−9	−22	−12
[190]	Rapeseed oil		RTFO PEN 70/100	SBS EOC			−87	−21			−72	85
[223]	Oakwood (W) Switchgrass (SG)	Pyrolysis	PG58-22 PG64-16	Polyethylene								
[228]	Waste engine oil		PG64-22	RAP								D_f_ −40.1

All values based on 5% BMB content, unless indicated. † Percent change in performance parameter (e.g., change in viscosity due to the addition of bio-binder. ‡ Performance for RTFO- and PAV-aged samples for rutting and fatigue resistances, respectively (DSR, MSR, or LAS results). * Viscosity of unaged bio-modified binder at 135 °C. ** Based on DSR (|G*|) and BBR (stiffness) tests results.

## Data Availability

Not applicable.

## Abbreviations

ABCD	Asphalt Binder Cracking Device
BA	Bagasse Ash
BBR	Bending Beam Rheometer
BBA	Bio-Based Asphalt (BBA)
BMB	Bio-Modified Asphalt Binder
BB	Bio-Oil/ Bio-Binder
BRA	Buton Rock Asphalt
SH	Castor Oil
DC	Corn Oil
CR	Crumb Rubber
DSO	Date Seed Oil
G*	Dynamic Shear Modulus
DSR	Dynamic Shear Rheometer
EFB	Empty Fruit Bunch
G*sinδ	Fatigue Parameter
TFTT	Fracture Toughness Temperature
HAP	Hard Asphalt Particles
HMA	Hot Mix Asphalt
IDT	Indirect Tensile Strength
LAS	Linear Amplitude Sweep
MSCR	Multiple Stress Creep Recovery
Jnr	Nonrecoverable Creep Compliance
PI	Penetration Index
PVN	Penetration Viscosity Number
PG	Performance Grade
BA	Petroleum-Based Asphalt (BA)
δ	Phase Angle
SBS	Poly(Styrene-Butadiene-Styrene
PE	Polyethylene
PMB	Polymer-Modified Bio-Oil
PPA	Polyphosphoric Acid
PAV	Pressure Aging Vessel
RP	Reactor Product
RAP	Reclaimed Asphalt Pavement
RAS	Reclaimed Asphalt Shingles
RA	Recycling Agents
RTFO	Rolling Thin Film Oven
RV	Rotational Viscosity
|G*|/sinδ	Rutting Resistance Parameter
SI	Segregation Index
SFA	Soy Fatty Acids
TSRST	Thermal Stress Restrained Specimen
TR	Tire Rubber Powder
TLA	Trinidad And Tobago
TPB	Trinidad Petroleum Asphalt Binder
UFO	Used Frying Oil
VECD	Visco-Elastic Continuous Damage
VTS	Viscosity–Temperature Susceptibility
WMA	Warm Mix Additives
WCO	Waste Cooking Oil
WEO	Waste Engine Oil
WVO	Waste Vegetable Oil
